# Psychophysiological responses to stride frequency manipulation in men and women

**DOI:** 10.1371/journal.pone.0355226

**Published:** 2026-08-05

**Authors:** Ribal Abdul Karim, Gavin Glasgow, Jill Baker, Jeannette Byrne, Rodrigo Hohl, Fabien A. Basset

**Affiliations:** 1 School of Human Kinetics and Recreation, Memorial University of Newfoundland, St. John’s, Newfoundland, Canada; 2 Department of Biophysics and Physiology, Institute of Biological Sciences, Federal University of Juiz de Fora (UFJF), Juiz de Fora, Brazil; NLA Hogskolen - Oslo, NORWAY

## Abstract

This study examined psychophysiological responses to stride frequency manipulation during walking in men and women. Nineteen healthy adults (11 men, 8 women) completed two sessions: familiarization session (session 1) during which the preferred stride frequency (PSF1) and its corresponding speed (PWS1) were determined, and experimental session (session 2) that consisted of participants walking at four 15-minute treadmill counterbalanced conditions: the preferred stride frequency measured in Session 1 and being imposed in session 2 called PSF2, new PSF measured in Session 2 (PSF3), and ±10% deviations from PSF2. Oxygen uptake (V̇O_2_), affective valence (Feeling Scale), arousal (Felt Arousal Scale), the rating of perceived exertion (RPE) and heart rate (HR) were measured. Repeated-measures ANOVA revealed significant main effects of stride frequency on V̇O_2_ (*p* < 0.001), feeling scale (*p* < 0.05), and RPE (*p* < 0.05). Planned contrasts showed that PSF3 elicited lower V̇O_2_ (*p* < 0.01, medium to large effect size), lower RPE (*p* < 0.05, medium to large effect size) and higher positive valence (*p* < 0.05, medium effect size) compared to the ± 10% deviations. Planned contrasts also showed that PSF3 compared to PSF2, elicited lower V̇O_2_ (*p* < 0.05, medium effect size), lower RPE (*p* < 0.05, medium effect size) and higher positive valence (*p* = 0.05, medium effect size). Heart rate recovery was faster in women (*p* < 0.05), but no sex-condition interactions emerged for any primary outcomes (all *p* > 0.05). Findings demonstrate that preferred stride frequency minimizes energy cost and RPE scores and reflects higher positive valence during walking with no difference between men and women in our sample.

## Introduction

Physical activity’s health benefits are well-documented in the literature [1,2], yet many people struggle to maintain regular exercise due to discomfort or negative affective experiences [3–6]. A key factor influencing exercise adherence resides in how people feel during physical activity and how exercise alters affective responses (pleasure or displeasure) and perceived exertion (RPE) [3–5,7,8]. These psychological responses may be shaped not only by exercise intensity but also by kinematic adjustments, such as changes in stride frequency. Indeed, they are highly sensitive to the interplay between external exercise parameters (i.e., stride frequency or speed) and internal biological states. For instance, it was demonstrated that internal biological variations, including differences between the luteal and follicular phases in eumenorrheic women, can significantly impair affect and motivation during high-intensity exercise, even when physiological markers remain unchanged [9]. This finding underscores the importance of considering both physiological parameters and individual constraints (i.e., potential sex-specific biological variations) when examining behavioral and psychological responses to altered movement patterns.

Humans naturally choose a walking stride frequency that minimizes energy cost, termed the preferred stride frequency (PSF) [10]. Stride frequency is primarily regulated by the central pattern generators (CPGs) [11,12]. Central pattern generators (CPGs) are neural circuits within the spinal cord that produce rhythmic motor patterns with minimal cortical input (central motor drive) [11,13]. While CPGs produce the basic locomotor rhythm, their function is continuously modulated by supraspinal input and somatosensory feedback, which become particularly important when the natural rhythm is disrupted [14–16]. Deviating from the PSF, whether by increasing or decreasing stride frequency, elevates energy cost in a U-shaped pattern [17–20].

Any departure from PSF is associated with increased heart rate and RPE [20]. While these physiological effects are well-documented, the impact on affective states, such as changes in pleasure or arousal, remains unclear. Imposed alterations in stride frequency may influence the conscious mental effort required to maintain neuromuscular drive in the limbs at a preferred walking speed. This, in turn, may affect perceived exertion and affective responses during exercise, as well as decision-making related to engaging in walking as parts of daily activities [21,22].

Moreover, the interplay between psychological and kinematic factors can modulate central command. Central command is a neural mechanism that synchronizes central motor drive (signals to muscles) with the cardiorespiratory efference (e.g., heart rate, respiratory rate) during exercise and contributes to shaping the perception of exertion [23,24]. According to this model, when motor patterns conflict with the body’s peripheral afferences feedback, central command must adjust motor output to align physiological strain with subjective feelings of effort, thereby enabling individuals to achieve their goal [23–25]. Accordingly, PSF may reflect the central command’s ability to select kinematic patterns that minimize both energy cost and psychological responses. Thus, deviations from PSF may disrupt the synergy between CPGs and central command, increasing conscious effort and forcing the central command to override a more efficient motor pattern. Consequently, pleasure may be reduced and energetic cost increases.

The aim of this study was to provide insight into the interplay between psychological and physiological responses to manipulated stride frequency. Specifically, as the primary outcome, we examined the effects of altering stride frequency on energy cost, affective response, and perceived exertion in an experimental sample of men and women. We also sought to determine whether walking at an imposed stride frequency elicits more unpleasant feelings than walking freely at one’s preferred stride frequency. As a secondary aim, we investigated whether men and women differ in their energetic and psychological responses to this manipulation. We hypothesized that the preferred stride frequency would elicit the lowest energy cost, the most favorable affective responses, and the lowest ratings of perceived exertion (RPE).

## Methodology

### Participants

The study was approved by the “Health Research Ethics Board (HREB)” at Memorial University of Newfoundland (HREB # 20250940 & HREB # 20251364) and was conducted in accordance with the Declaration of Helsinki. All participants provided written informed consent in the familiarization session prior to data collection. Nineteen healthy individuals (11 men and 8 women) aged 18–35 years were recruited from the general population of Newfoundland, Canada.

Participants were screened for their physical activity level and any history of cardiopulmonary, metabolic, or orthopedic conditions using the Canadian Society for Exercise Physiology (CSEP) *Get Active Questionnaire* and were excluded if they answered “yes” to any question. Additionally, participants were excluded if they did not achieve a “fair” score for their age group on the $\dot{V}$O_2max_ test, according to American College of Sports Medicine guidelines [26], or if their body mass index (BMI) exceeded 30.

### Protocol

#### Overview.

The study was conducted in the “Laboratory of Exercise and Environmental Physiology” at the School of Human Kinetics and Recreation at Memorial University of Newfoundland. Throughout the experiments, interaction with participants and cell phone use were avoided to mitigate significant affective interference. In addition, to control for shoe weight and heel-to-toe drop effects, participants were provided cushion socks. Moreover, to mitigate exercise discomfort participants were asked to wear a T-shirt and shorts. Lastly, to control for any effect of diurnal metabolic fluctuation, each session took place between 7:00 and 12:00 in the morning. A 2-hour and 20-minute commitment was required from the participant to complete the 2 sessions (1 familiarization and 1 experimental).

#### Session 1.

The first session consisted of explaining the experimental procedures, obtaining the consent form and collecting the Get Active Questionnaire (GAQ) and demographic information. Height and weight were collected using an electronic scale and a stadiometer, respectively. Afterwards, participants were asked to lie down in bed for 15 minutes to mitigate any significant affective interference with the lab setting prior to recording resting HR and baseline psychometrics measurements [Feelings Scale (FS) and Felt Arousal Scale (FAS)] [27].

Next, preferred walking speed (PWS1) and its corresponding stride frequency (PSF1) were determined by asking participants to walk for 15 minutes at a speed that was most comfortable to them. Participants were instructed to adjust the speed during the first 5 minutes and then to walk at the selected speed for the remaining 10 minutes, during which PSF1 was determined. To help participants better self-select their PWS1 the following instructions were given: “While you are walking today, imagine that it is the first beautiful spring day, and you decide to take a walk outside. You are not walking for fitness, just for leisure, so you can go as fast or as slow as is comfortable for you” [28].

**Fig 1:**
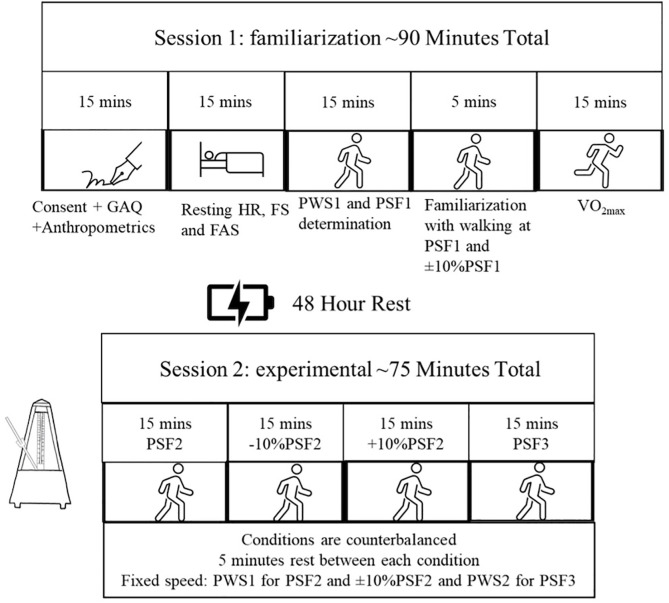
Experimental protocol. The first session (called familiarization session) consisted of HR, FS and FAS baseline measurements in addition to determination of preferred walking speed (PWS1) and stride frequency (PSF1), familiarization with imposed stride frequencies (at PSF1, at 10% below PSF1 and at 10% above PSF1) using a metronome and determination of the maximal oxygen uptake. Session 2 (called experimental session) consisted of four counterbalanced conditions: three of which were imposed using a metronome (at PSF2, at 10% below PSF2 and at 10% above PSF2), and the remaining condition was walking at their preferred stride frequency (PSF3) and pace (PWS2) with the same instructions as session 1. The imposed conditions were performed at the speed corresponding to the pace they themselves chose during session 1 (PWS1).

To familiarize participants with the experimental session (session 2), a 5-minute practice period was implemented at PWS1 after the 15-minute walk. Participants were asked to adjust to a metronome beat set at three frequencies: PSF1 and 10% above and below*.* Participants could not see any information from the treadmill panel during the walking in sessions 1 and 2. A 48-hour washout period between the two sessions was implemented to reduce the carryover effect of exercise (See Fig 1).

#### Session 2.

Participants randomly completed four 15-minute walking conditions, each separated by a 5-minute rest period. The four conditions were as follows:

1. PSF2 – The metronome was set to the preferred stride frequency determined during Session 1 (PSF1).
2. +10%PSF2 – The stride frequency was set to 10% greater than PSF2.
–10%PSF2 – The stride frequency was set to 10% less than PSF2.
4. PSF3 – Participants freely selected their stride frequency using the same protocol as in Session 1.

Although PSF2 matched the frequency of PSF1, it represented a constrained walking condition because participants had to synchronize with the PSF1 metronome beats while complying with the treadmill speed. PSF3 was implemented to ensure any potential biological variation. Therefore, PSF3 represents a truly *self-selected*, natural gait pattern free from external timing constraints. This distinction allows us to disentangle the effects of the frequency *value* from the effects of the constraint *itself.*

During all experimental sessions, heart rate, oxygen uptake and walking kinematics were recorded throughout the conditions. Psychometric parameters (RPE, FS, FAS) were assessed at 0-, 3-, 6-, 9-, 12- and 15-minute marks during walking and 3-minute post-exercise.

### Women-specific methodology

To control for the potential confounding effects of the menstrual cycle, all female participants were scheduled to complete their sessions during the follicular phase or the non-active phase. To determine their cycle phase, participants first completed a Menstrual Cycle Survey. A woman researcher then used this survey to identify the start of the follicular phase. Participants were also asked whether they consistently tracked their cycle using an application or calendar to help verify that their cycle length was within the 21- to 32-day criteria. If a participant did not track her cycle, the research team used a backward count method based on the projected start date of her next menses [29].

Participants could be naturally menstruating (menstrual cycle length ≥21 days and ≤ 32 days), using oral contraceptives in a cycling formulation or using a copper IUD. Those using oral contraceptive in a cycling formulation (21–24 days in active phase, 4–7 days in non-active phase) were asked to complete the experiment during the inactive (non-hormonal) phase, as this relates to the follicular phase in naturally menstruating women. Participant were excluded if pregnant, experiencing any disruptions in their menstrual cycle due to illness or disease (polycystic ovary syndrome, endometriosis etc.), experiencing irregular menstrual cycles (more than 32 days, less than 21 days, absence of menstrual cycle), or taking hormonal replacement therapy (such as skin patches, implants, vaginal creams, etc.).

### Psychometric measures

#### Affective response.

Affective responses were assessed using the circumplex model, according to which affect is defined by the dimensions of affective valence (pleasure-displeasure) and perceived activation arranged within the area of the circle defined by the two dimensions [30]. The affective valence was assessed using the Feelings Scale (FS), which consists of an 11-point rating scale ranging from +5 to −5, with anchor points at zero (“Neutral”) and all odd integers, from “Very Good” (+5) to “Very Bad” (−5) [31]. Activation was assessed using the Felt Arousal Scale (FAS), a six-point rating scale ranging from 1 to 6, with anchor points corresponding to “Low Arousal” (1) and “High Arousal” (6) [32]. The various affective states are conceived as combinations of feeling and arousal in different degrees.

Affective responses are evaluated as ordinal data. Trespassing into quadrants and proximity to the circle perimeter are used as references for qualitative analysis. To maintain analytical parsimony when examining core affect during exercise within the circumplex model, we report the mean from 3 to 15 minutes and its 95% confidence interval (95% CI) on the orthogonal axis.

#### Rating of perceived exertion (RPE).

Rating of perceived exertion scale was used to measure participants’ rating of perceived exertion during walking [33]. Participants were asked to rate their subjective level of effort on a 6–20 points Likert scale where 6 means “very, very light” and 20 “very, very hard”, reflecting feelings of physical stress and fatigue. For participants to differentiate diffuse somatic exertion (i.e., interoceptive sensory signals generated by the peripheral organs) from mental effort (i.e., feedforward drive) to maintain lower limb ambulation [21,34], they were asked “how do you perceive the difficulty in keeping your legs active at this moment?”.

### Kinematic parameters

All participants were videorecorded while walking using a video camera (Sony ZV-1M2) positioned sagittal to the treadmill to shoot the side of body below the waist, with no audio collected. A sampling rate of 120 Hz was used. To facilitate the determination of stride frequency, a reflective marker was placed on the right malleolus and on the side of the treadmill. Video files were saved using participant ID codes and stored on a secure laboratory drive.

PSF1 determination consisted of averaging the number of strides (i.e., right heel strike to right heel strike) counted in three non-overlapping 60-second windows sampled within the 10-minute steady-state period: recorded minutes 6–7 (beginning of steady-state), 10–11 (middle), and 14–15 (end). For each condition in session 2, stride counts were obtained from three non-overlapping 60-second windows placed early, mid and late in the 15-minute bout to avoid start-up transients and overlap with psychometric sampling: recorded-minutes 1–2 (beginning), 7–8 (middle), and 13–14 (end).

Stride counting was performed by two researchers who inspected the video using Kinovea video analysis and modeling tool software (version 2023.1.2, Charmant, 2023). Each researcher performed counts independently and without knowledge of the other’s counts; the final stride count for each 60-second window was taken as the mean of the two researchers’ counts.

### Physiological measurement

#### Heart rate (HR) and heart rate recovery.

Heart rate was wirelessly transmitted to a computer using a heart rate monitor (Polar Monitor, model H10) during all sessions. Mean heart rate was averaged into 1-minute blocks for the incremental test and 5-minute blocks for the four different walking conditions. In addition, heart rate recovery was computed for 1 minute immediately after the four 15-minute walking blocks in each experimental session.

#### Indirect calorimetry: Energy cost.

An indirect calorimetry system (Sable Systems International, Las Vegas, NV, USA) was used to measure oxygen uptake (V̇O2) and carbon dioxide production (V̇CO_2_) during each of the walking conditions. The system was set to record the fractional amount of oxygen and carbon dioxide, mixing chamber temperature, water vapor pressure, barometric pressure, subsample flow rate, and mass flow rate in a negative pressure design. The mass flow generator and controller (FK-500) were set at a rate of 200 L min^-1^ for exercise metabolic rate. A subsample of that flow (sub-sampler, SS4) pulled at 150 ml min^-1^ through a water vapor analyzer (RH-300), a dual infrared carbon dioxide analyzer, and a paramagnetic oxygen analyzer (CA-10 Carbon Dioxide and PA-10 Oxygen Analyzers). Fractions of gasses in the room were recorded before and after each measurement for baseline references. Prior to testing, the oxygen and carbon dioxide analyzers were calibrated with room air and reference gasses (100% nitrogen and 1% carbon dioxide). Water vapor pressure was zeroed after drying sample gasses by passing through a column of magnesium perchlorate and the sub-sampler pump was calibrated using a flow meter (Gilmont Rotameter). Gas volumes included in metabolic calculations were to be expressed at standard conditions of temperature, pressure, and dry from water (STPD) [35].

### Statistical analysis

Statistical analyses were performed using IBM SPSS Statistics (version 29.0.2.0., NY, USA). The normality of data was verified using Shapiro-Wilk’s test and visual inspection of the Q-Q plots and histograms. Levene’s test was used to assess the homogeneity of variance. Mauchly’s test was applied to check the sphericity of variance. When the sphericity of variance was violated, Huynh–Feldt correction was applied to adjust the significance of *p*-values and degrees of freedom.

Although the Feeling Scale, Felt Arousal Scale, and ratings of perceived exertion (RPE) yield ordinal data, they were analyzed using parametric methods. This approach is consistent with established practice for psychophysiological and affective scales in exercise science [5,31] and with evidence that parametric tests are robust when applied to multi-category ordinal scales [36].

Independent-samples *t*-tests were used to compare anthropometrics and physical activity variables between men and women. In addition, paired-samples *t*-tests were used to compare the stride frequencies of the conditions as a manipulation check to confirm that the stride frequencies during the experimental conditions differed as intended.

A three-way mixed-design ANOVA, (4 conditions x 5 times x 2 sexes) was conducted on FS, FAS, RPE and heart rate during walking (from minute 3–15). Secondly, a two-way mixed-design ANOVA, (4 conditions x 2 sexes) was conducted on the energy cost during walking and on the heart rate recovery. Decomposing any significant interactions or differences between condition, time, and sex was performed using within-subject planned contrasts, which are appropriately powered with the current sample to test our a priori hypotheses. Based on our theoretical framework that the truly self-selected stride frequency (PSF3) represents a psychophysiological optimum, we had three specific comparisons of interest: (1) PSF3 vs. −10%PSF2, (2) PSF3 vs. + 10%PSF2, and (3) PSF3 vs. PSF2. This focused set of contrasts was chosen over omnibus post-hoc tests (e.g., Bonferroni) to maximize statistical power for detecting the effects of theoretical interest and to minimize the risk of Type II error associated with correcting for multiple comparisons that are not central to our hypotheses. To address the possibility of inflated Type I error across the planned contrasts, we additionally applied a Benjamini-Hochberg false-discovery-rate (FDR) correction to the contrasts within each primary outcome as a sensitivity analysis; FDR-adjusted *p*-values are reported in S1 Table.

To minimize the small-sample bias N = 19 < 20, Hedges’*g* correction was applied to Cohen’s *d*_*z*_ according to the equation *g* = *J* × *d*_*z*_, in which *J* =$\;\text{1}-\;\frac{\text{3}}{\text{4}(N-\text{1})-\text{1}}=\;\frac{\text{3}}{\text{4}(\text{1}\text{9}-\text{1})-\text{1}}$ = 0.96 [37–39]. Statistical significance was set at *p ≤* 0.05. Data were reported as mean ± standard deviation (SD).

## Results

### Participants’ anthropometrics

The participants’ anthropometrics are shown in Table 1. Participants were young (25 ± 5 years), physically active (228 ± 161 minutes/week of moderate-intensity aerobic physical activity) and had a BMI of 24 ± 5. The independent samples *t*-test showed that when compared to men, women differed significantly in weight (< 21%), height (< 8%) and had lower body weight, *t*_(17)_ = 2.335, *p* < 0.05, and were shorter, *t*_(17)_ = 7.422, *p* < 0.001.

**Table 1:** Participants’ characteristics. Men (N = 11), women (N = 8).

Variable	Sex	Mean	SD	95% Confidence Interval	
				Lower Bound	Upper Bound
Age (years)	Men	26	4	23	29
	Women	22	5	18	27
	All	25	5	22	27
Weight (kg)	Men	79 *	19	66	92
	Women	62	9	54	70
	All	72	17	63	80
Height (cm)	Men	177**	3	175	180
	Women	163	5	158	167
	All	171.	8	167	175
Physical activity (minutes/week of moderate-intensity aerobic physical activity)	Men	198	142	103	293
	Women	270	186	115	425
	All	228	161	151	306
BMI	Men	25	6	21	29
	Women	23	4	20	26
	All	24	5	22	27

Mean ± SD. *and ** denote a statistically significant difference between men and women. *p < 0.05, **p < 0.001.

### Kinematics

The stride frequency outcomes are presented in Table 2. The dependent samples *t*-test showed that PSF3 significantly differed from −10%PSF2, *t*_(18)_ = 7.256, *p* < 0.001 and +10%PSF2, *t*_(18)_ = 6.553, *p* < 0.001. Fur*t*hermore, −10%PSF2 differed from +10%PSF2 by 9 strides per minute. Finally, stride frequency (Δ = 1, *p* > 0.1) and speed (i.e., PWS1 and PWS2 = ~ 3 km/h, Δ = 0.02, *p* > 0.78) did not differ significantly between PSF2 and PSF3.

**Table 2:** Stride frequencies in the four conditions.

Conditions	Strides/minute	Delta
−10%PSF2†	45 ± 4	4 ± 4*
PSF3	49 ± 4	0
+10%PSF2†	54 ± 5	5 ± 3*
PSF2	50 ± 4	1 ± 2

Mean ± SD. * Denotes a statistically significant difference between ±10%PSF2 and PSF3. † Denotes a statistically significant difference between ±10%PSF2 and PSF2. *p* < 0.001. Note that delta differences are based on PSF3.

### Energy cost

The two-way ANOVA (4 conditions x 2 sexes) was performed on energy cost and revealed no significant condition x sex interaction (*F*_(3,51)_ = 0.610, *p* = 0.612) and no main significant effect of sex (*F*_(1,17)_ = 1.076, *p* = 0.314). However, a significant main effect of condition was found, (*F*_(3,51)_ = 7.632, *p* < 0.001).

**Fig 2:**
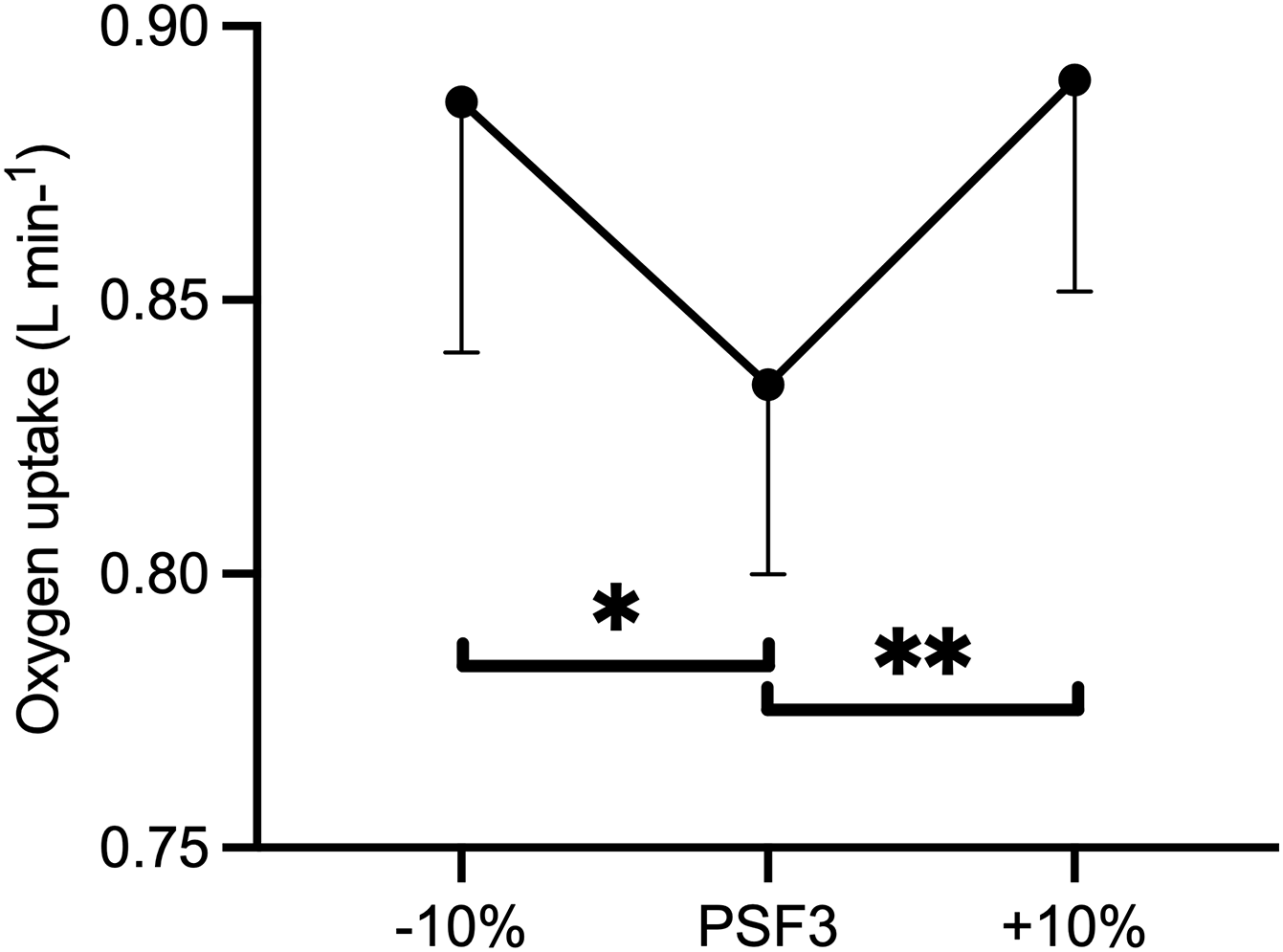
Energy cost of walking conditions. * Denotes a statistically significant difference between Preferred Stride Frequency 3 and −10%Preferred Stride Frequency 2 (*p* < 0.01). ** Denotes a statistically significant difference between Preferred Stride Frequency 3 and +10% Preferred Stride Frequency 2 (*p* < 0.001).

As displayed in Fig 2, planned contrasts analysis showed that the oxygen uptake for PSF3 (0.83 ± 0.15 L/min) was significantly lower than for −10%PSF2 (0.88 ± 0.19 L/min; *F*_(1,17)_ = 9.408, *p* = 0.007) and for +10%PSF2 (0.89 ± 0.16 L/min; *F*_(1,17)_ = 25.272, *p* < 0.001). The effect sizes for −10%PSF2 (*d*_*z*_ *=* 0.70, *g* = 0.67) and for +10%PSF2 (*d*_*z*_ = 1.15, *g* = 1.10) reached a medium to large magnitude with a strong correlation between stride frequency and energy cost (*r* = 0.59 and *r* = 0.77, respectively). In addition, the energy cost at PSF3 (0.83 ± 0.15 L/min) was significantly lower than at PSF2 (0.86 ± 0.17 L/min; *F*_(1,17)_ = 5.072, *p* = 0.038) with a medium effect size (*d*_*z*_ *=* 0.51, *g* = 0.49) and a medium correlation (*r* = 0.48). Although, PSF2 and PSF3 showed the same stride frequency and walking speed, PSF2 energy cost did not significantly differ from −10%PSF2 and +10%PSF2.

After Benjamini-Hochberg correction, all planned contrasts for this outcome remained significant (FDR-adjusted *p* ≤ 0.04; S1 Table).

### RPE

**Fig 3:**
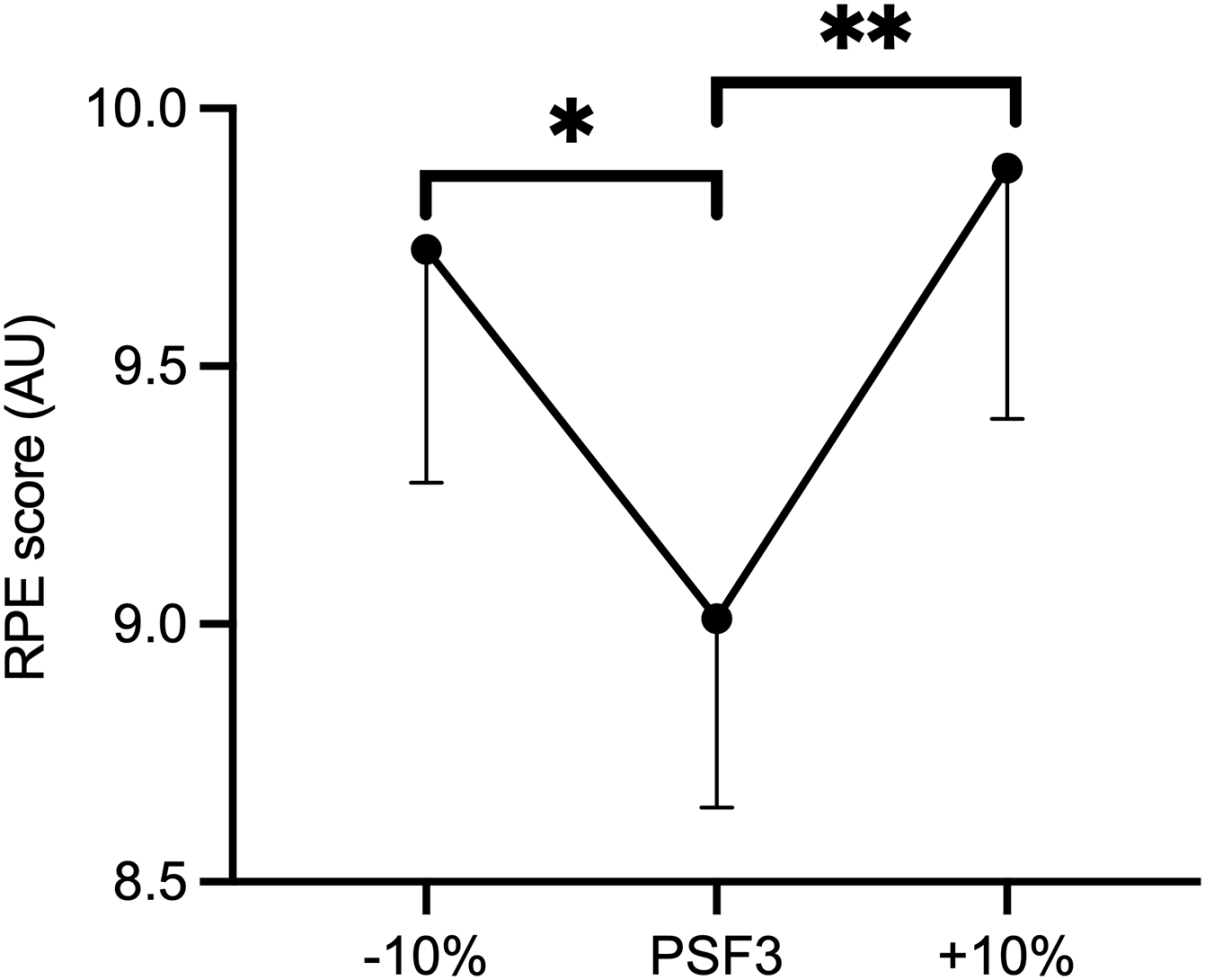
Rate of perceived effort of walking conditions. * Denotes a statistically significant difference between Preferred Stride Frequency 3 and −10% Preferred Stride Frequency 2. **Denotes a statistically significant difference between Preferred Stride Frequency 3 and +10% Preferred Stride Frequency 2. *p* ≤ 0.01.

A three-way ANOVA (4 conditions x 5 times x 2 sexes) was conducted to detect any significant effect of factors on RPE (See Fig 3). No significant interaction was found between condition x time x sex (*F*_(8.143,138.428)_ = 0.986, *p* = 0.450), between condition x sex (*F*_(3,51)_ = 0.937, *p* = 0.430), between condition x time (*F*_(8.143,138.428)_ = 0.603, *p* = 0.704) and between time x sex (*F*_(4,68)_ = 0.200, *p* = 0.937). No main effect of sex (*F*_(1,17)_ = 1.311, *p* = 0.268) was revealed. However, a significant main effect of conditions was found (*F*_(3,51)_ = 3.321, *p* = 0.027), as well as a significant main effect of time (*F*_(1.524, 25.901)_ = 16.349, *p* < 0.001).

Decomposing the conditions’ main effect, planned contrasts analysis showed that PSF3 (8.95 ± 0.37) elicited a significantly lower RPE compared to −10%PSF2 (9.63 ± 0.44; *F*_(1,17)_ = 8.461, *p* = 0.01) and to +10%PSF2 (9.78 ± 0.48; *F*_(1,17)_ = 13.144, *p* = 0.002) (Fig 3). The effect sizes for −10%PSF2 (*d*_*z*_ = 0.66, *g* = 0.63) and for +10%PSF2 (*d*_*z*_ = 0.83, *g* = 0.79) reached a medium to large magnitude with a strong correlation between stride frequency and RPE (*r* = 0.57 and *r* = 0.66, respectively). In addition, RPE during PSF3 (8.95 ± 0.37) was significantly lower than during PSF2 (9.52 ± 0.40; *F*_(1,17)_ = 5.047, *p* = 0.038) with a medium magnitude (*d*_*z*_ = 0.51, *g* = 0.49) and a medium to large correlation *(r* = 0.48). Although, PSF2 and PSF3 showed the same stride frequency and walking speed, RPE during PSF2 did not significantly differ from −10%PSF2 and +10%PSF2.

After Benjamini-Hochberg correction, all planned contrasts for this outcome remained significant (FDR-adjusted *p* ≤ 0.04; S1 Table).

Planned contrasts analysis showed that RPE significantly increased at all time points (*p* < 0.05). RPE scores were 8.72, 9.31, 9.52, 9.85, and 9.96 for 3, 6, 9, 12, and 15 minutes, respectively.

### Feeling Scale and Felt Arousal Scale

**Fig 4:**
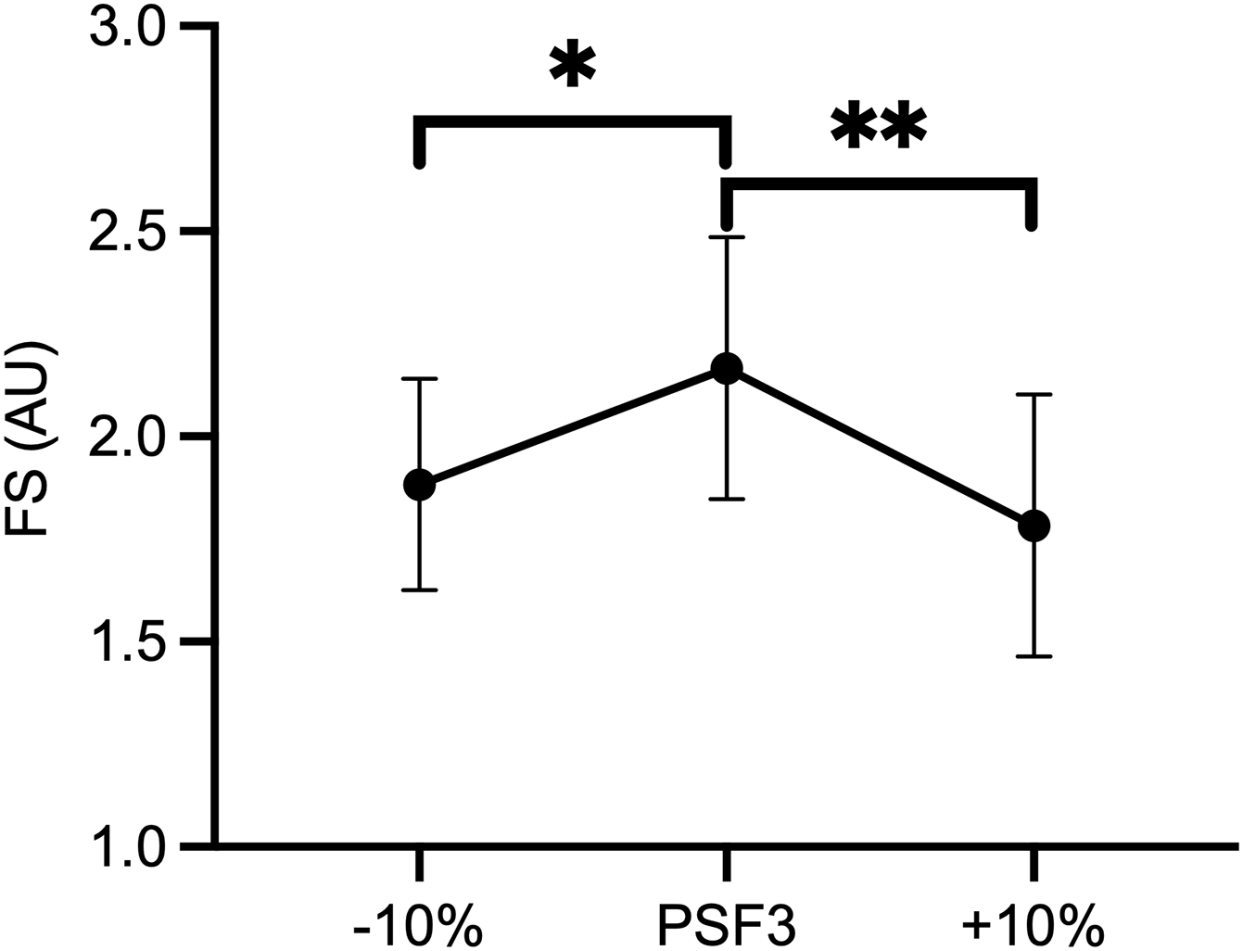
Feeling scale of walking conditions. * Denotes a statistically significant difference between Preferred Stride Frequency 3 and −10% Preferred Stride Frequency 2. ** Denotes a statistically significant difference between Preferred Stride Frequency 3 and +10% Preferred Stride Frequency 2. *p* < 0.05.

The three-way ANOVA (4 conditions x 5 times x 2 sexes) revealed no significant interaction between condition x time x sex (*F*_(6.560,111.524)_ = 1.074, *p* = 0.384), between condition x sex (*F*_(3,51)_ = 0.593, *p* = 0.622), between condition x time (*F*_(6.560,111.524)_ = 1.307, *p* = 0.216) and between time x sex (*F*_(4,68)_ = 1.886, *p* = 0.123). There was also no significant main effect of condition (*F*_(3,51)_ = 2.637, *p* = 0.060), sex (*F*_(1,17)_ = 0.587, *p* = 0.454) or time (*F*_(4,68)_ = 1.167, *p* = 0.333). Since sex did not significantly interact with condition or time, data from women and men were pooled together to boost the statistical power, to increase the residual degree of freedom and to simplify the model by reducing the number of factors. Then, a two-way ANOVA was performed and revealed a main effect of condition (*F*_(3,54)_ = 2.942, *p* = 0.041). Planned contrasts analysis revealed that PSF3 (2.179 ± 0.37) elicited a significant positive pleasure compared to −10%PSF2 (1.726 ± 0.30; *F*_(1,18)_ = 6.089, *p* = 0.024) and to +10%PSF2 (1.768 ± 0.36; *F*_(1,18)_ = 5.318, *p* = 0.033) (Fig 4). The effect sizes for both −10%PSF2 (*d*_*z*_ = 0.56, *g* = 0.53) and +10%PSF2 (*d*_*z*_ = 0.53, *g* = 0.50) reached a medium magnitude and a medium to large correlation between condition and pleasure (*r* = 0.50 and *r* = 0.47, respectively). Positive pleasure in PSF3 (2.179 ± 0.37) was significantly higher compared to PSF2 (1.926 ± 0.38; *F*_(1,18)_ = 4.412, *p* = 0.05) with a medium magnitude (*d*_*z*_ = 0.48, *g* = 0.46) and medium correlation (*r* = 0.44). Although, PSF2 and PSF3 showed the same stride frequency and walking speed, the pleasure experienced in PSF2 did not significantly differ from pleasure in −10%PSF2 and +10%PSF2.

After Benjamini-Hochberg correction, the planned contrasts for pleasure remained significant at the conventional threshold but were close to the boundary (FDR-adjusted *p* ≈ 0.05; S1 Table), indicating this effect is less robust than the metabolic and perceptual effects.

Moreover, for FAS, the three-way ANOVA (4 conditions x 5 times x 2 sexes) revealed no significant interaction between condition x times x sexes (*F*_(12,204)_ = 0.284, *p* = 0.991), between condition x sex (*F*_(3.51)_ = 0.311, *p* = 0.817), between condition x time (*F*_(12,204)_ = 1.379, *p* = 0.178) and between time x sex (*F*_(1.611,27.380)_ = 0.186, *p* = 0.785). No significant main effect of condition (*F*_(3,51)_ = 0.271, *p* = 0.846), sex (*F*_(1,17)_ = 0.014, *p* = 0.907) or time (*F*_(1.611,27.380)_ = 0.306, *p* = 0.692) was observed (See Fig 4).

### Circumplex model outcomes

**Fig 5:**
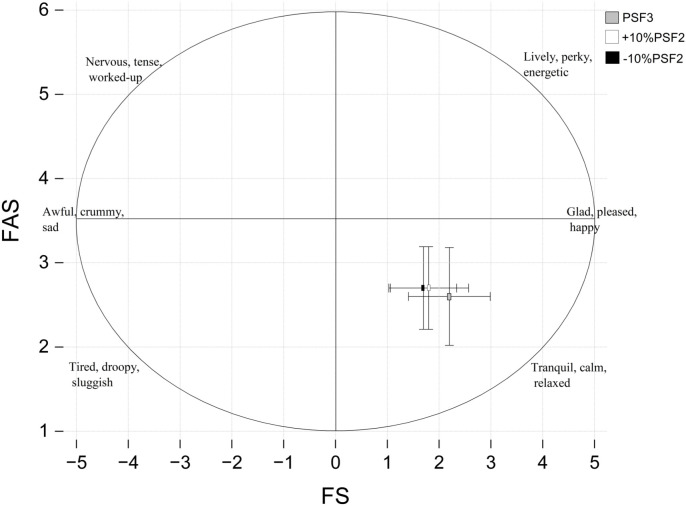
Circumplex model. The mean core affect (95% CI) for all conditions (Preferred Stride Frequency 3 and ±10% Preferred Stride Frequency 2) was maintained in the lower-right quadrant (low activation, positive valence), indicating a tranquil and calm state.

A mixed quali-quantitative analysis of core affect, based on the circumplex model (See Fig 5) and its orthogonal association between FAS and FS, revealed that all conditions consistently maintained the mean (95%CI) affect state within the lower right quadrant (low activation and positive valence). Fig 5 also shows that the PSF3 condition resulted in a more tranquil/calm core affect compared to the ± 10% PSF2 conditions.

### Heart rate

A three-way ANOVA (4 conditions x 5 times x 2 sexes) was conducted on HR that revealed no significant interaction between condition x times x sexes (*F*_(3.237,55.036)_ = 1.446, *p* = 0.237), condition x sex (*F*_(3,27.036)_ = 3.092, *p* = 0.072), between condition x time (*F*_(3.237,55.036)_ = 1.514, *p* = 0.219) and between time x sex (*F*_(2.457,41.764)_ = 0.438, *p* = 0.689). No significant main effect of condition (*F*_(1.590,27.036)_ = 3.355, *p* = 0.060), sex (*F*_(1,17)_ = 0.212, *p* = 0.651) or time was observed (*F*_(2.457,41.764)_ = 2.126, *p* = 0.122).

### Heart rate recovery

As for heart rate recovery, the two-way ANOVA (4 conditions x 2 sexes) showed no significant condition x sex interaction (*F*_(3,51)_ = 1.072, *p* = 0.369) and no main significant effect of condition (*F*_(3,51)_ = 1.359, *p* = 0.266). However, a significant main effect of sex was found on heart rate recovery (*F*_(1,17)_ = 6.344, *p* = 0.022) where women had significantly higher heart rate recovery (19.25 ± 4.10 beats/minute for women versus 15.34 ± 2.68 beats/minute for men, respectively).

## Discussion

This study aimed to investigate the effect of stride frequency manipulation on psychophysiological responses during walking in men and women. We hypothesized that walking at the preferred stride frequency would result in the lowest energy cost and perceived exertion, along with greater positive affect, compared to imposed stride frequencies in both sexes. The findings confirm that the preferred stride frequency represents a psychophysiological optimum characterized by reduced energy cost and effort, and enhanced pleasure with no significant differences between men and women.

This study reinforces the well-established U- or V-shaped relationship between stride frequency and energy cost [10,17–19]. This relationship suggests that the brain naturally selects a preferred stride frequency that minimizes energy cost for a given walking speed [10,17–19]. The observed 6–9% increases in energy cost and RPE at ±10% deviations indicate that even small departures from this optimal pattern impose high physiological and perceptual costs, likely due to disruption of the neurophysiological locomotor control system, particularly the communication between higher cortical motor control areas and the CPG.

Although rhythmic auditory stimulation may facilitate locomotor automaticity [40], our findings – increased oxygen uptake, higher perceived exertion, and reduced pleasure – suggest that imposing a ± 10% stride frequency deviation with a metronome acted as a significant stressor, thereby disrupting the automaticity. The control of walking is characterized by a dynamic balance between automatic and executive processes that depends on the specific demands of the task [40]. Forcing individuals to walk at imposed frequencies likely increased the biomechanical and cognitive demands [41]. Consequently, continuously monitoring and synchronizing steps with the metronome relied on an attention-demanding dual task [40]. Because automatic processing is far less sensitive to stressors than is performance under controlled/executive processing [42], this forced compensatory reliance on top-down executive resources may explain not only the elevated oxygen uptake but also the increased perceived effort and the pleasure decline. Additionally, imposed stride frequencies can amplify afferent feedback from proprioceptors, signaling to the central nervous system that the movement is suboptimal [43]. This heightened afferent input, combined with the increased executive demand to override the preferred gait pattern, likely exacerbates RPE scores.

It is also important to consider alternative, biomechanical explanations for our findings, which are not mutually exclusive with the neurophysiological mechanisms discussed above. Deviating from the preferred stride frequency inevitably alters joint kinematics and muscle activation patterns. For instance, walking at a slower frequency may require greater knee and hip flexion to maintain speed, while a faster frequency may increase the work of the ankle plantar-flexors [19]. Such changes can alter the mechanical efficiency of the muscles, affecting the length-tension and force-velocity relationships, which would directly impact energy cost and, subsequently, RPE [18]. These observations align with the concept of resonance tuning, where the preferred frequency of rhythmic movement corresponds to the resonant frequency of the muscle-limb system, minimizing the mechanical effort required to sustain motion [44]. When movement frequency deviates from this resonant state, additional muscular force is needed to overcome the system’s natural dynamics, contributing to elevated energy demand. Similarly, Kuo [45] demonstrated through biomechanical modeling that humans select step lengths and frequencies that minimize metabolic cost, particularly by reducing the force/time demands on muscles. Deviations from this optimized pattern necessarily increase energy cost. Furthermore, the need to continuously correct foot placement to match a metronome could impose a subtle but cumulative energetic cost, independent of the frequency itself, arising from the cognitive and neuromuscular demands of auditory-motor synchronization [46–48], which may have contributed to the differences observed between PSF2 and PSF3.

Notably, this study detected significant increases in energy cost and RPE even at smaller deviations (±10% or approximately 4–5 strides/minute) than those reported previously [20], a study that found no significant changes at such small deviations, likely due to their smaller all-male sample (n = 11). While the absolute differences in RPE scores between conditions were relatively small, the concomitant shifts in affective valence were meaningful. This aligns with the findings of Rose et al. [49], who demonstrated that even a subtle increase in exercise intensity-corresponding to a 4% difference in %HRmax-was sufficient to shift an individual’s affective experience from feeling “good” (FS + 3) to only “fairly good” (FS + 1). In the present study, the slightly elevated RPE in the imposed conditions (PSF2, −10%PSF2, + 10%PSF2) was associated with a significant reduction in pleasure compared to the truly self-selected PSF3 condition. This dissociation underscores the distinction between the two constructs, as articulated in the dual-mode model [5,50]: RPE reflects the sensory dimension of “what” one is experiencing (the strain and heaviness of work), while the Feeling Scale captures the affective interpretation of “how” that experience makes one feel [5,31,49]. These findings have practical implications for exercise prescription, as even small increases in perceived effort-when imposed rather than self-selected-may diminish the valence of the experience, potentially undermining long-term adherence [4,5,8].

Interestingly, the higher energy cost and RPE scores observed in the present study occurred a lower walking speed (~ 3 km/h) compared to previous studies, which reported speeds of 5.4 km/h [17], 4.6 km/h [19] and 4.7 km/h [20]. However, these increases did not compromise participants’ ability to complete the 15-minute walking task, as indicated by positive pleasure ratings and low arousal, reflecting a calm/tranquil core affect within the circumplex model. According to Venhorst et al. [22], deterioration in core affect, manifested as reduced pleasure/valence and increased arousal, typically precedes the onset of an action crisis, an intrapsychic conflict between goal pursuit and disengagement that undermines volitional control and commitment. In this context, the calm/tranquil core affect observed across all conditions suggests that walking for 15 minutes in a non-preferred stride frequency in a controlled laboratory setting does not trigger such a crisis. This outcome, however, might differ if the imposed conditions were sustained for a longer duration.

Our findings on the Feeling Scale align with the broader literature on the relationship between exercise intensity and core affect, which indicates that higher intensity is generally associated with lower pleasure [5,7,8,51]. Previous studies by Ekkekakis and Lind [7] and Lind et al. [8] reported decreased positive feelings when preferred walking speed was increased by 10% in sedentary, middle-aged women living with obesity. The present study extends this understanding by demonstrating that walking stride frequency and affective response appear to follow a similar pattern to that observed for walking speed. While our exploratory analysis revealed a significant effect of stride frequency on Feeling Scale when data were pooled, further research with larger samples and greater statistical power is needed to confirm its impact on pleasure.

A compelling finding of this study was that, despite identical stride frequency between PSF2 and PSF3, PSF2 resulted in a 4% increase in energy cost, a 6% rise in RPE score, and a 12% reduction in pleasure compared to PSF3. This affective outcome underscores the critical role of perceived autonomy in shaping the exercise experience. As highlighted by Rose and Parfitt [49], when individuals are allowed to self-regulate their exercise intensity, they report a more pleasant affective experience compared to when an intensity is imposed, even if the physiological load is similar (near ventilatory threshold). In the context of the dual-mode model [50], during low-intensity exercise (e.g., below or around the ventilatory threshold), affective responses appear to be less influenced by associative cognitive processes, including the appraisal of the exercise experience [49,52]. Accordingly, the imposed, metronome-paced condition (PSF2) likely disrupted the cognitive appraisal, requiring participants to continuously monitor and correct their gait to match an external cue. This additional cognitive load may have been interpreted as a threat to autonomy or a source of frustration, thereby attenuating the positive affective response typically associated with self-selected movement [5]. In contrast, PSF3 allowed for a “natural” gait, fostering a sense of control and a more positive appraisal, reflected in the higher Feeling Scale scores. This interpretation aligns with Vazou-Ekkekakis and Ekkekakis [53] who attributed reduced pleasure in imposed-intensity exercise to diminished perceived autonomy.

The dissociation between PSF2 and PSF3 underscores the central origin of the perceived effort, as described by the corollary discharge model [54]. PSF2 represents a constrained condition because the metronome-imposed stride frequency required continuous conscious interference with CPGs. Although not directly measured, this pattern remains consistent with the notion that perceived exertion under imposed pacing primarily reflects central neural drive rather than solely peripheral strain [34,55–57]. Specifically, PSF2 perhaps demanded ongoing conscious error correction mediated by the executive control and supplementary motor area to synchronize CPGs with the metronome [40,46,58], thereby increasing activation of central command regions [46,48]. This additional cortical load likely amplified corollary discharge signals, directly elevating RPE scores [54]. These frequent adjustments likely imposed a subtle but cumulative energetic cost, which may explain the increased oxygen uptake and RPE observed in PSF2 compared to PSF3, despite identical stride frequencies and walking speed.

Furthermore, given that PSF2 and PSF3 likely produced the same peripheral afferent signals due to their identical stride frequency, we postulate that the observed differences in RPE scores and positive affect may be attributed to increased brain metabolic demand. Functional MRI studies of paced finger-tapping show that auditory pacing – comparable to the metronome used in our study – preferentially activates the bilateral ventral premotor cortex, supplementary motor area, cerebellar hemispheres, and thalamus [48]. These regions are critical for internal timing, error monitoring, and corrective adjustments [46], factors that likely played a significant role in perceived effort during PSF2 compared with PSF3.

### Methodological considerations and future directions

First, the relatively small sample size, consisting of young, healthy, and physically active individuals, limits generalizability. Future research should include more diverse populations (e.g., older adults, sedentary individuals, and clinical groups) to assess the universality of these results. Second, we fully acknowledge that the between-sex comparisons in this study are underpowered. With 11 men and 8 women, the minimal detectable between-group effect (Cohen’s d) with 80% power and α = 0.05 is approximately 1.38; the power to detect a medium effect (d = 0.5) is only about 17%. Thus, the absence of statistically significant sex differences in our sample should be interpreted cautiously and not as evidence of equivalence. Future studies with larger and more balanced samples are required to test sex differences reliably.

Third, treadmill walking, while offering experimental control, may not fully replicate overground locomotion. Subsequent studies could incorporate detailed neurophysiological measures and examine overground walking for extended durations beyond 15 minutes. Additionally, poorer affect and lower motivation during the luteal compared to the follicular phase were reported in eumenorrheic women, particularly at higher exercise intensities, despite no physiological differences [9]. Future research should investigate whether stride frequency manipulations elicit different psychophysiological responses across menstrual cycle phases. To better control for menstrual cycle phases, future studies may benefit from incorporating hormonal assays to confirm menstrual phase. Although we employed rigorous screening and scheduling methods (including the backward count method recommended by Schmalenberger et al. [29], some degree of phase misclassification remains possible.

Moreover, although affective and perceptual scales were analyzed parametrically in line with common practice, their ordinal nature remains a limitation.

## Conclusion and practical application

Our findings demonstrate that the preferred stride frequency represents an optimal point for energy cost, RPE and pleasure during walking. Even small deviations from this preferred pattern significantly increase energy cost, reduce pleasure, and elevate perceived effort. These results underscore the importance of considering individual movement preferences in exercise prescription and suggest that allowing self-selection of stride frequency may enhance the psychological experience of walking and, ultimately, support long-term physical activity adherence. Evidence shows that when adults self-select their pace, they naturally gravitate towards the heart rate and oxygen uptake ranges recommended for the development and maintenance of cardiorespiratory health [59]. Thus, in practice, self-regulation of gait does not preclude achieving physiological overload; rather, it may help individuals find a sustainable frequency and intensity.

## Supporting information

S1 Table Planned contrast statistics for the primary outcomes, with Benjamini-Hochberg false-discovery-rate (FDR) adjusted *p*-values. (DOCX)
