# Supplementary material for: Psychophysiological responses to stride frequency manipulation in men and women
Source: PLoS One. 2026 Aug 5;21(8):e0355226. doi: 10.1371/journal.pone.0355226 (PMC13440853; doi:10.1371/journal.pone.0355226)
Supplement: Supplementary file 1 [file pone.0355226.s001.docx]

**S1 Table.** Planned contrast statistics for the primary outcomes, with Benjamini-Hochberg false-discovery-rate (FDR) adjusted *p*-values.

*Contrasts compare the freely self-selected condition (PSF3) against the imposed conditions. V̇O₂ and RPE contrasts are from the repeated-measures model (df = 1, 17); Feeling Scale contrasts are from the sex-pooled model (df = 1, 18). d_z_ = Cohen’s d for within-subject (paired) designs; g = Hedges’ g; r = effect-size correlation.*

| **Outcome** | **Contrast** | **F** | **df** | ***p* (raw)** | **d_z_** | **g** | **r** |
| --- | --- | --- | --- | --- | --- | --- | --- |
| **Oxygen uptake (V̇O₂)** | | | | | | | |
| V̇O₂ | PSF3 vs +10%PSF2 | 25.272 | 1, 17 | <0.001 | 1.15 | 1.10 | 0.77 |
|  | PSF3 vs -10%PSF2 | 9.408 | 1, 17 | 0.007 | 0.70 | 0.67 | 0.59 |
|  | PSF3 vs PSF2 | 5.072 | 1, 17 | 0.038 | 0.51 | 0.49 | 0.48 |
| **Rating of Perceived Exertion (RPE)** | | | | | | | |
| RPE | PSF3 vs +10%PSF2 | 13.144 | 1, 17 | 0.002 | 0.83 | 0.79 | 0.66 |
|  | PSF3 vs -10%PSF2 | 8.461 | 1, 17 | 0.010 | 0.66 | 0.63 | 0.57 |
|  | PSF3 vs PSF2 | 5.047 | 1, 17 | 0.038 | 0.51 | 0.49 | 0.48 |
| **Feeling Scale (affective valence)** | | | | | | | |
| FS | PSF3 vs -10%PSF2 | 6.089 | 1, 18 | 0.024 | 0.56 | 0.53 | 0.50 |
|  | PSF3 vs +10%PSF2 | 5.318 | 1, 18 | 0.033 | 0.53 | 0.50 | 0.47 |
|  | PSF3 vs PSF2 | 4.412 | 1, 18 | 0.050 | 0.48 | 0.46 | 0.44 |

**FDR-adjusted p-values (Benjamini–Hochberg, within each outcome family of three contrasts):**

| **Outcome** | **Contrast** | ***p* (raw)** | **p (FDR)** |
| --- | --- | --- | --- |
| V̇O₂ | PSF3 vs +10%PSF2 | <0.001 | <0.001 |
|  | PSF3 vs -10%PSF2 | 0.007 | 0.011 |
|  | PSF3 vs PSF2 | 0.038 | 0.038 |
| RPE | PSF3 vs +10%PSF2 | 0.002 | 0.006 |
|  | PSF3 vs -10%PSF2 | 0.010 | 0.015 |
|  | PSF3 vs PSF2 | 0.038 | 0.038 |
| FS | PSF3 vs -10%PSF2 | 0.024 | 0.049 |
|  | PSF3 vs +10%PSF2 | 0.033 | 0.049 |
|  | PSF3 vs PSF2 | 0.050 | 0.050 |

**Note.** After Benjamini–Hochberg FDR correction, all V̇O₂ and RPE contrasts remained significant (adjusted *p* ≤ .038). The Feeling Scale contrasts remained significant at the conventional α = .05 threshold but lay close to the boundary (adjusted *p* ≈ .05), indicating that the affective valence effect, while present, is of more modest robustness than the metabolic and perceptual effects and is interpreted accordingly in the main text.
