# Supplementary material for: Psychophysiological responses to stride frequency manipulation in men and women
Source: PLoS One. 2026 Aug 5;21(8):e0355226. doi: 10.1371/journal.pone.0355226 (PMC13440853; doi:10.1371/journal.pone.0355226)
Supplement: Supplementary file 2 [file pone.0355226.s002.docx]

**Major Comments & Questions**

1. **Data Availability Compliance**

**Question:** Your Data Availability Statement currently claims data is available "upon reasonable request" due to ethics restrictions. *PLOS ONE* policy explicitly prohibits "data on request" unless legal/ethical restrictions are insurmountable.

Can you please provide a de-identified, anonymized "minimal data set" (e.g., a spreadsheet with individual participant values for VO2, RPE, FS, FAS)?

If your ethics board strictly forbids *any* public sharing of anonymized data, can you provide the direct contact information for the Ethics Committee (not an author) to whom data requests can be sent, along with the specific text of the ethical restriction?

2. **Statistical Power and Sex Differences**

**Question:** You conclude that there are "no differences between men and women" based on a sample of 11 men and 8 women. Given the small sample size, is this finding a result of true physiological equivalence or a Type II error (lack of statistical power)?

**Action:** Can you provide a post-hoc power analysis to demonstrate whether your study had sufficient sensitivity to detect a medium or small effect size between sexes?

If not, please rephrase your conclusion to state that "no differences were detected" rather than asserting equivalence, and discuss this limitation.

3. **Mechanistic Speculations (Central Command & Autonomy)**

**Question:** In the Discussion, you attribute the increased RPE and reduced pleasure in the imposed condition (PSF2) to "diminished perceived autonomy" and "increased central command/error monitoring".

Since you did not measure autonomy (e.g., via questionnaire) or cortical activity (e.g., EEG/fMRI), what specific data supports these claims?

**Action:** Can you revise the discussion to frame these explanations as *hypotheses* rather than definitive conclusions?

Alternatively, do you have ancillary data to support these mechanistic assertions?

4. **Familiarization Protocol Adequacy**

**Question:** Participants were given only a "5-minute practice period" to familiarize themselves with metronome-guided walking.

How did you determine that 5 minutes was sufficient to overcome the "cognitive load" of synchronizing steps with a beat?

Could the elevated RPE in the metronome conditions be a result of acute task novelty rather than the stride frequency itself?

**Minor Comments & Clarifications**

**Figure Error Bars**

**Question:** In Figures 2, 3, and 4, do the error bars represent Standard Deviation (SD) or Standard Error of the Mean (SEM)? This is not specified in the captions. Please clarify and update the captions accordingly.

5. **Statistical Precision**

**Question:** In Table 1, BMI is reported as an integer (e.g., "25") while other anthropometric variables are reported with decimals.

Can you standardize the reporting of data precision to one decimal place for consistency?

6. **Language and Phrasing**

**Question:** In the Methods (Line 101), the phrase "interaction... were refrained" is grammatically incorrect.

Did you mean "interaction was minimized" or "participants refrained from interaction"? Please clarify.

7. **Menstrual Cycle Verification**

**Question:** You mention using a "backward count method" for women who did not track their cycle.

How did you account for cycle variability in these participants to ensure they were truly in the follicular phase?

Were any hormonal assays (e.g., progesterone levels) used to validate this phase assignment?
