# Supplementary material for: Psychophysiological responses to stride frequency manipulation in men and women
Source: PLoS One. 2026 Aug 5;21(8):e0355226. doi: 10.1371/journal.pone.0355226 (PMC13440853; doi:10.1371/journal.pone.0355226)
Supplement: Supplementary file 3 [file pone.0355226.s004.docx]

We thank the Academic Editor and the three reviewers for their careful reading and constructive suggestions which improved the manuscript substantially. Below we respond point-by-point to each comment and describe the changes made in the revised manuscript. Page/line numbers below refer to the tracked-changes manuscript.

# Response to Journal Requirements and Editor's Comments

**Journal Requirement 1 (PLOS ONE Style):** We have thoroughly reviewed the PLOS ONE formatting guidelines and have ensured that our manuscript, including file naming, adheres to the required style.

**Journal Requirement 2 (Data Availability):** The study protocol has been approved by the Health Research Ethics Board, T: +1 709 864 8871, F: 709 864 8870, [www.hrea.ca](http://www.hrea.ca).

To comply with PLOS ONE’s data policy while adhering to the ethical constraints regarding participant privacy, the underlying data set has been deposited at Borealis Memorial University Dataverse. To protect participant anonymity as mandated by our ethics approval, the data are held under restricted access. Interested researchers who meet the criteria for access to confidential data may apply for access through [<https://doi.org/10.5683/SP3/2ANOJ3>], subject to approval by the Health Research Ethics Board and the signing of a Data Use Agreement.

We remain available to address any further questions the editorial board or reviewers may have regarding our submission.

**Major Comments & Questions**

1) **Data availability compliance:** answered in the previous comment.
2) **Statistical Power and Sex Differences:** That is a fair and critical question. We answered this also in the response to Reviewer #2.

We fully acknowledge this critical limitation. Our conclusion of "no differences between men and women" was stated too strongly given our sample size. We have revised the language throughout the manuscript to reflect that we simply did not detect a significant difference in this sample, rather than asserting equivalence. Regarding a post-hoc power analysis, we have carefully considered this point. Methodological literature strongly advises against traditional post-hoc power calculations (Hoenig & Heisey, 2001) as they are mathematically redundant with the *p*-value and can be misleading for data interpretation. As a more informative alternative, we conducted a post-hoc sensitivity analysis. With n=11 (men) and n=8 (women), the minimal detectable Cohen's d (80% power, two-sided α = 0.05) is ≈ 1.38, and the power to detect a medium effect (d = 0.5) is ≈ 17%. These values quantify the limited sensitivity of our between-sex comparisons.

**Actions taken:**

a) Abstract: it has been revised (Page 2, Line 31): “...with no significant differences detected between men and women in our sample.”
b) Discussion: it has been revised and the following was added in page 24, lines 514-519: “Second, we fully acknowledge that between-sex comparisons in this study are underpowered. With 11 men and 8 women, the minimal detectable between-group effect (Cohen's d) with 80% power and α = 0.05 is approximately 1.38; the power to detect a medium effect (d = 0.5) is only about 17%. Thus, the absence of statistically significant sex differences in our sample should be interpreted cautiously and not as evidence of equivalence. Future studies with larger and balanced samples are required to test sex differences reliably”.
c) Made clear in the “Statistical Analysis” page 12, lines 251-259, that the study's primary tests were the within-subject planned contrasts, which are appropriately powered and form the basis of our main conclusions.

We believe these revisions appropriately contextualize the null sex findings while maintaining scientific rigor.

3) **Mechanistic Speculations (Central Command & Autonomy):** This is an excellent point. We responded also to the same comment provided by Reviewer #2.

We agree that our initial discussion framed these mechanisms too definitively. We have now extensively revised the Discussion to frame these explanations as *plausible hypotheses* derived from the literature, not as conclusions directly proven by our data. We have also added a consideration of alternative biomechanical explanations (as suggested by Reviewer #3).

Actions taken:

a) Elaborated our interpretation of the RPE and FS findings. Thus, we added two paragraphs: a paragraph (page 20-21, lines 433-447) elaborating on the small difference in RPE and the relationship between RPE and pleasure, and another paragraph (page 22-23, lines 473-488) elaborating on the dissociation between PSF2 and PSF3.
b) Toned down speculative language: we used “may be attributed to” (line 493) and “perhaps” (line 494).
c) We have also added a consideration of alternative biomechanical explanations (as suggested by Reviewer #3) on pages 19-20, lines 410-429.
d) Removed the paragraph detailing neuron excitability, which was on page 19, lines 394-403 in the original manuscript.
4) **Familiarization Protocol Adequacy:** We thank the reviewer for this important methodological question. Our rationale for considering this sufficient was based on three factors:
a) Simplicity of the task**:** Synchronizing footsteps with an isochronous metronome at a self-selected walking speed is a relatively low-complexity motor task. Research on sensorimotor synchronization indicates that the basic ability to match steps to a beat is readily accessible to healthy adults, even without extensive practice (Repp & Su, 2013).
b) Prior exposure during PSF1 determination: Before the familiarization period with the metronome, participants had already walked for 15 minutes at their preferred speed while their natural stride frequency was measured. Thus, by the time they were exposed to the metronome, they were already accustomed to the treadmill and the walking speed.
c) Pilot testing: In pilot work preceding this study, we observed that pilot participants who have never been exposed to a metronome before readily matched the metronome frequency and could achieve basic synchronization within 2–3 minutes. The 5-minute period was therefore chosen to provide ample time for all participants to become comfortable.

However, we fully agree with the reviewer that task novelty cannot be entirely ruled out as a contributing factor to the elevated RPE in the metronome conditions.

**Minor Comments & Clarifications**

1) **Figure Error Bars:** We thank the reviewer for noting this. The error bars in Figures 2, 3, and 4 represent Standard Deviation (SD).  We have updated the figure captions accordingly to specify this.
2) **Statistical Precision:** We thank the reviewer for this attention to detail. We have revised Table 1 to report all continuous variables (including BMI) with integers. We decided to use integers and not one decimal place for scientific accuracy for example for age and BMI as someone cannot be for instance 23.4 years old.
3) **Language and Phrasing:** We thank the reviewer for catching this grammatical error. The correct sentence is “interaction with participants and cell phone usage **was** refrained”. Correction was made.
4) **Menstrual Cycle Verification:** We thank the reviewer for this important question regarding menstrual cycle verification. We acknowledge that precise phase confirmation ideally requires hormonal assays, and the absence of such assays is a limitation of our study. We added this limitation in pages 24-25, lines 527-530.

However, we implemented several methodological controls to maximize the accuracy of phase assignment within the constraints of our study design:

a) Screening for cycle regularity: Only women with self-reported regular menstrual cycles (21–32 days) were included. Those with irregular cycles, polycystic ovary syndrome, endometriosis, or other conditions affecting cycle regularity were excluded.
b) Verification methods:

- For women who tracked their cycle using apps or calendars (n = 5), we used their tracking data to confirm follicular phase timing.
- For those who did not track (n = 3), we used the backward count method based on the projected start date of their next menses, as recommended by Schmalenberger et al. (2020) for situations where hormonal assays are not feasible.

c) Scheduling window: All sessions were scheduled within days 1–7 of the follicular phase (or the inactive phase for oral contraceptive users), which is the most reliable window for identifying early follicular phase without hormonal confirmation.
d) Confirmation at session start: At the beginning of each session, participants were asked to confirm they were menstruating or in the expected phase based on their tracking or backward count.

We remain available to address any future questions or edits requested by the editorial board or reviewers regarding our submission.

**Additional Editor Comments:** We have provided a point-by-point response to each of the three expert reviewers below.

We remain available to address any future questions or edits requested by the editorial board or reviewers regarding our submission.

# Response to Reviewers' Comments

## Reviewer #1

**1- General Comment:** “it is not clear why PSF3 condition was used as a landmark condition to identify the influence of stride frequency on the measured variables. I think that the following comparisons may be useful for this study: PSF2 versus PSF2+10%, PSF2 versus PSF2-10%, and PSF2 versus PSF3. Furthermore, I think that oxygen uptake can be normalized to lean body mass in this study.”

**1- Our Response:** we thank the reviewer for this important observation, as it highlights a critical aspect of our experimental design that we have now clarified in the manuscript. PSF3 is the true, unconstrained preferred stride frequency. The rationale for using PSF3 as the reference is that it represents a completely self-selected, natural motor pattern, free from any external constraint.

In contrast, PSF2, while numerically identical to PSF1, is an *imposed* condition where participants must consciously synchronize their steps with a metronome. This introduces a cognitive load that is absent during natural walking. Therefore, comparing the imposed conditions (-10%PSF2, +10%PSF2, PSF2) against the truly natural condition (PSF3) allows us to isolate the specific effect of an externally imposed constraint from the effect of the stride frequency value itself. We got inspired by Vazou-Ekkekakis & Ekkekakis, 2009 [study mentioned in the discussion section, Vazou-Ekkekakis, S., & Ekkekakis, P. (2009). Affective consequences of imposing the intensity of physical activity: does the loss of perceived autonomy matter? *Hellenic Journal of Psychology*, *6*, 125–144.] that reported that imposed self-selected exercise intensity, as opposed to self-selected intensity, reduces pleasure.

Thus, because that contrast (PSF3 vs PSF2 and PSF3 vs ±10%PSF2) was pre-specified, we used planned contrasts (resulted from the 3-way mixed design ANOVA) with PSF3 as the reference.

Action taken: we have added a clearer explanation on page 8, lines 147-149 and page 12-13, lines 251-259.

Furthermore, regarding oxygen uptake being normalized to lean body mass, we believe that normalizing oxygen uptake by lean body mass does not bring further information into this investigation.

2- **Specific Comments:**

A- **Reviewer’s comment**: “**Title:** I think that the title of the article can be revised for more clarity. For example, I think that the ‘Psychophysiological responses’ can be changed to ‘Metabolic cost and perceptual responses’.”
A- **Our Response:** we thank the reviewer for this suggestion. However, to ensure terminological precision, we have opted for a slightly different revision. While "perceptual responses" typically refers to sensory experiences like perceived effort, our study also measured affective responses (pleasure and arousal) using the Feeling Scale and Felt Arousal Scale. Therefore, to accurately encompass all our measured psychological variables (both perceptual and affective), and to keep the terminological consistency for “energy cost”, we prefer keeping the title as it is.
B- **Reviewer’s comment: “Abstract:**
1) I think that the ‘psychophysiological responses’ can be changed (e.g., metabolic cost and perceptual responses).
   2) I think that the ‘perceived effort (RPE)’ should be revised to ‘rating of perceived exertion (RPE)’.”
B- **Our response:**
1) Same response as (A) above
2) We thank the reviewer for this suggestion. However, to ensure terminological precision, we differentiate between “effort” and “exertion”. As mentioned in the Methodology section page 10, lines 193-196, based on (Abbiss et al., 2015; Venhorst et al., 2018a), “Exertion” is considered to refer to the peripheral fatigue (i.e., interoceptive sensory signals generated by the peripheral organs) while “Effort” is considered to refer to the mental fatigue (i.e., feedforward drive) to maintain lower limb ambulation (the sense of how hard one is trying). [Abbiss, C. R., Peiffer, J. J., Meeusen, R., & Skorski, S. (2015). Role of Ratings of Perceived Exertion during Self-Paced Exercise: What are We Actually Measuring? *Sports Medicine (Auckland, N.Z.)*, *45*(9), 1235–1243. <https://doi.org/10.1007/S40279-015-0344-5> , Venhorst, A., Micklewright, D., & Noakes, T. D. (2018a). Towards a three-dimensional framework of centrally regulated and goal-directed exercise behaviour: a narrative review. *British Journal of Sports Medicine*, *52*(15), 957–966. <https://doi.org/10.1136/bjsports-2016-096907>]
C- **Reviewer’s comment: “Introduction:**

1) I think that ‘psychological responses’ can be changed (e.g., perceptual responses).

2) I think that more detailed information regarding the ‘central pattern generators’ can be provided.”

C- **Our Response:**

1) Same response as (A) above
2) We added more details about central pattern generators on page 3-4, lines 54-57.
D- **Reviewer’s comment: “Methodology:**
1) What is the scientific rationale for the determination of the duration (i.e., 15 min)?
   2) I think that the ‘Rating of Perceived Effort’ can be changed to ‘Rating of Perceived Exertion’.”

D- **Our Response:**

1) The 15-minute duration was chosen to allow participants to reach a physiological steady state and to provide sufficient time to observe the evolution of psychological responses similar to the following article: Ekkekakis, P., Hall, E. E., & Petruzzello, S. J. (2008). The relationship between exercise intensity and affective responses demystified: to crack the 40-year-old nut, replace the 40-year-old nutcracker! *Annals of Behavioral Medicine : A Publication of the Society of Behavioral Medicine*, *35*(2), 136–149. https://doi.org/10.1007/S12160-008-9025-Z
2) Same response as (B) above.
E- **Reviewer’s comment: “Results:**

1) Please double-check the accuracy of the statistical methods (e.g., The dependent samples t-test (Page 13, Lines 260~265)).

2) It is not clear why PSF3 (not PSF2) condition was used to identify the influence of stride frequency on the measured variables.

E- **Our Response:**

1) We have double-checked these analyses. The dependent samples t-tests were used specifically to compare the stride frequencies between conditions (e.g., PSF3 vs. -10%PSF2) to confirm our experimental manipulation was successful. This is a manipulation check, separate from the main ANOVA analyses for the dependent variables. To clarify, we have revised the sentence to be more precise on page 12, lines 246-247.
2) Same response as in our response to general comments above.
F- **Reviewer’s comment: “Discussion:**

1) I think that most of the Discussion section is based upon speculations. I think that the article would be stronger if the authors could focus more on their research outcomes (e.g., relatively small difference in rating of perceived exertion). Furthermore, I think that the following article may possibly be useful for the interpretation/discussion regarding oxygen uptake and perceptual responses:

・Rose and Parfitt. Medicine and Science in Sports and Exercise, 40(10), 1852–1860, 2008.

2) I think that it may be interesting to present each participant’s felt arousal scale data.

3) I think that the information regarding the central pattern generators (Page 18, Lines 379~386) can be moved to the Introduction section.

4) I think that the ‘small sample size’ may not be related to the ‘generalizability’ (Page 22, Lines 460~461).

5) I think that this sentence (Page 22, Lines 473~475) can be revised (e.g., ‘small’)

F- **Our Response:**

1) This is a critical and fair point. We thank the reviewer for the reference (Rose & Parfitt, 2008). This paper provides useful context on the relationship between affect and perceived exertion. Actions taken:

- We have cited this paper in the Discussion to support our interpretation of the RPE and FS findings. Thus, we added two paragraphs: a paragraph (page 20-21, lines 433-447) elaborating on the small difference in RPE and the relationship between RPE and pleasure, and another paragraph (page 22-23, lines 473-488) elaborating on the dissociation between PSF2 and PSF3.
- Tone down speculative language: we used “may be attributed to” (line 493) and “perhaps” (line 494).
- We have also added a consideration of alternative biomechanical explanations (as suggested by Reviewer #3) on pages 19-20, line 410-429.
- Removed the paragraph detailing neuron excitability, which was on page 19, lines 394-403 in the original manuscript.

2) While we agree that individual data can be informative, presenting 19 individual time-series for four conditions would be visually overwhelming. The circumplex model in Figure 5 provides a concise summary of the combined FS and FAS data, showing that all conditions remained in the "calm/tranquil" quadrant.
3) We have moved the detailed description of CPGs and their role to the Introduction.
4) We respectfully disagree. A small, homogenous sample (young, healthy, active) directly limits the generalizability of the findings to other populations (e.g., older adults, clinical populations).
5) That is a fair point. However, we kept the word “small” for term consistency throughout the paper, for example “smaller deviations” at line 430, “small deviations” at line 432 and “small departures” at line 390.
G- **Reviewer’s comment: “Figures:**

1) I think that the y-axis of the Figures can be corrected (e.g., I think that rating of perceived exertion can be presented from 6 to 20).”

G- **Our Response:**

1) We thank the reviewer for this suggestion to improve the clarity of the figure. Our analysis focuses on the relative differences between conditions (the U-shaped relationship), thus, we believe that presenting the full range of the Borg RPE scale does not provide any additional insight into our result.

We remain available to address any future questions or edits requests by the editorial board or reviewers regarding our submission.

## Reviewer #2

A- **Reviewer’s Comment:** “Unsupported Primary Conclusions (Statistical Power)
   A central conclusion of the manuscript is that there are "no differences between men and women". However, with a sample size of only 8 women and 11 men, the study is severely underpowered for between-subjects comparisons. The failure to detect a significant difference is highly likely a Type II error rather than proof of equivalence. Basing a primary conclusion on an underpowered null finding is a fatal flaw in the analysis.”
A- **Our Response:** We fully acknowledge this critical limitation. Our conclusion of "no differences between men and women" was stated too strongly given our sample size. We have revised the language throughout the manuscript to reflect that we simply did not detect a significant difference in this sample, rather than asserting equivalence. Regarding a post-hoc power analysis, we have carefully considered this point. Methodological literature strongly advises against traditional post-hoc power calculations (Hoenig & Heisey, 2001) as they are mathematically redundant with the *p*-value and can be misleading for data interpretation. As a more informative alternative, we conducted a post-hoc sensitivity analysis. With n=11 (men) and n=8 (women), the minimal detectable Cohen's d (80% power, two-sided α = 0.05) is ≈ 1.38, and the power to detect a medium effect (d = 0.5) is ≈ 17%. These values quantify the limited sensitivity of our between-sex comparisons.

**Action taken:**

a) Abstract: it has been revised (Page 2, Line 31): “...with no significant differences detected between men and women in our sample.”
b) Discussion: it has been revised and the following was added in page 24, lines 514-519: “Second, we fully acknowledge that between-sex comparisons in this study are underpowered. With 11 men and 8 women, the minimal detectable between-group effect (Cohen's d) with 80% power and α = 0.05 is approximately 1.38; the power to detect a medium effect (d = 0.5) is only about 17%. Thus, the absence of statistically significant sex differences in our sample should be interpreted cautiously and not as evidence of equivalence. Future studies with larger and balanced samples are required to test sex differences reliably”.
c) Made clear in the “Statistical Analysis” page 12, lines 251-259, that the study's primary tests were the within-subject planned contrasts (resulted from the 3-way mixed design ANOVA), which are appropriately powered and form the basis of our main conclusions.

We believe these revisions appropriately contextualize the null sex findings while maintaining scientific rigor.

B- **Reviewer’s Comment:** “Extreme Over-Interpretation of Mechanisms
   The discussion relies heavily on speculative mechanisms to explain the differences between the PSF2 and PSF3 conditions. Attributing the increased perceived effort and decreased pleasure to "diminished perceived autonomy," "central command," and "increased brain metabolic demand" is entirely unsubstantiated by the data. The study includes no neurophysiological (e.g., EEG, fMRI) or psychological autonomy measures to support these claims, making the discussion highly conjectural rather than data-driven.”

B- **Our Response**: This is an excellent point. We agree that our initial discussion framed these mechanisms too definitively. We have now extensively revised the Discussion to frame these explanations as *plausible hypotheses* derived from the literature, not as conclusions directly proven by our data. We have also added a consideration of alternative biomechanical explanations (as suggested by Reviewer #3).

Actions taken:

a) Elaborated our interpretation of the RPE and FS findings. Thus, we added two paragraphs: a paragraph (page 20-21, lines 433-447) elaborating on the small difference in RPE and the relationship between RPE and pleasure, and another paragraph (page 22-23, lines 473-488) elaborating on the dissociation between PSF2 and PSF3.
b) Toned down speculative language: we used “may be attributed to” (line 493) and “perhaps” (line 494).
c) We have also added a consideration of alternative biomechanical explanations (as suggested by Reviewer #3) on pages 19-20, lines 410-429.
d) Removed the paragraph detailing neuron excitability, which was on page 19, lines 394-403 in the original manuscript.
C- **Reviewer’s Comments:** “Violation of Data Availability Policy
   The manuscript currently violates PLOS ONE’s strict Open Data policy. Stating that data is only available "from the corresponding author upon reasonable request" due to ethics restrictions is not acceptable. For a manuscript to be considered, authors must either provide the minimal anonymized dataset or provide the direct contact information of an ethics committee or legal body that fields data requests.”

C- **Our Response:** The study protocol has been approved by the Health Research Ethics Board, T: +1 709 864 8871, F: 709 864 8870, [www.hrea.ca](http://www.hrea.ca).

To comply with PLOS ONE’s data policy while adhering to the ethical constraints regarding participant privacy, the underlying data set has been deposited at Mendeley Data. To protect participant anonymity as mandated by our ethics approval, the data are held under restricted access. Interested researchers who meet the criteria for access to confidential data may apply for access through [Link/DOI], subject to approval by the Health Research Ethics Board and the signing of a Data Use Agreement.

D- **Reviewer’s Comment:** Limited Generalizability
   The small sample size (N=19) of strictly young, healthy, and physically active individuals severely limits the generalizability of the findings, particularly given the broad claims made about human locomotion and exercise adherence. Given the fundamental issues with statistical power and the speculative nature of the discussion, the manuscript is not suitable for publication in its current form.”

D- **Our Response:** We agree and have already addressed this point in our response to the first comment and in our revisions to the Discussion’s Methodological Considerations and Future Directions section. We remain available to address any future questions or edits requests by the editorial board or reviewers regarding our submission.

## Reviewer #3

A- **Reviewer’s Comment:** “Major Concerns
   1. I am concerned about type I error rate in the study. Multiple paired t-tests are used to compare stride frequencies without a clear rationale for not using ANOVA. Also planned comparisons are used to compare all combinations of conditions when post hocs would be appropriate. Planned comparisons should be reserved for specific hypotheses made in the paper and should not compare all combinations.
A- **Our Response:** We appreciate the opportunity to clarify our statistical approach, which was chosen to align with our specific *a priori* hypotheses.
1) Stride Frequency Comparisons: The paired t-tests were used solely as manipulation checks to verify that our experimental conditions resulted in significantly different stride frequencies (e.g., PSF3 vs. -10%PSF2). This is a standard and acceptable use of multiple t-tests for confirming that our manipulation was successful, as it is not part of the hypothesis testing for the dependent variables.
2) Planned Contrasts for Dependant Variables: We used the planned contrasts resulted from the 3-way mixed design ANOVA for our analyses because they were driven by *a priori* hypotheses. We had three specific comparisons of interest: PSF3 vs. -10%PSF2, PSF3 vs. +10%PSF2, and PSF3 vs. PSF2. As we have explained in the response to **Reviewer #1**, PSF3 was our theoretical "optimal" reference. A post-hoc test like Bonferroni would have compared all six possible pairwise combinations, including ones with no theoretical basis (e.g., -10%PSF2 vs. +10%PSF2). This would have increased the risk of Type II error (failing to find a true effect) by correcting for multiple comparisons that were not of primary interest. Our approach of using three planned contrasts with a per-comparison alpha of 0.05 is statistically valid and more powerful for our specific, pre-planned questions. We have clarified this rationale in the manuscript. Actions taken:
a) We have revised the sentence to be more precise on page 12, line 246 regarding the paired samples *t*-tests as a manipulation check.
b) We have added a clearer explanation on page 8, lines 147-149.
c) We have expanded our explanation in the statistical Analysis section, page 12, lines 251-259.
B- **Reviewer’s Comment:** “Major Concerns

2. The findings are interpreted in terms of changes to central command and motoneuron excitability, however, no consideration has been made for the biomechanical changes that occur with walking at different stride frequencies. There is extensive literature that considers how the biomechanics of the body impact PSF (see references below for examples). I would at least expect the authors to consider alternative explanations although of course use their results to support their claims.”

B- **Our Response:** This is an excellent and valid point. Our initial discussion was indeed focused on neurophysiological mechanisms.

We have now revised the Discussion to include a consideration of biomechanical factors on pages 19-20, lines 410-429.

**Reviewer’s Comment:** Minor Concerns: “

Abstract

The conclusion that PSF minimizes psychological responses is not accurate. The results show it resulted in higher positive valence.
Introduction
Line 44: Why is altering stride frequency not a change in exercise intensity? Don’t the findings of previous research show that walking at non-PSF increases intensity as indicated by oxygen consumption, heart rate (HR), and rating of perceived exertion (RPE)?
Method
Line 94: When and what protocol was used to determine VO2max?
Line 194: According to a quick online search, the Sony ZV-1M2 has a maximum video frame rate of 120 Hz. Please check and confirm what was used in the experiment.
Line 239: Why were paired samples t-test used to assess stride frequency? It’s not clear why 3-way mixed design ANOVA was not used. Repeated uses of paired samples t-tests increase type I error rate. Also, be specifying “within conditions” I was expecting just comparisons within conditions but the results section seems to included comparisons between conditions.
Line 244: Why were planned contrasts used instead of post hoc tests? Planned comparisons inflate the experiment-wise type I error rate and are therefore used to address specific hypotheses and should not be used to “decompose any significant interactions or differences”. Post hoc tests are designed to test all combinations of conditions and can control experiment-wise error rate, depending on type used.
Results
Line 255: The claim that women were significantly leaner does not match the Table 1. According to the table values the two gender groups fit firmly within each other’s 95% confidence intervals.
Line 281: Specify, the interpretation of effect size magnitude in the method section. I believe Cohen would conclude that r=.48 is medium (<.5).
Discussion
Line 377: It is not clear that changing stride frequency by 10% disrupts CPGs and the locomotor control system. My understanding of central pattern generators (CPGs) is that they can readily produce a range of frequencies. What evidence is there that CPGs have a specific frequency that matches the preferred stride frequency of walking? Increased physical and perceptual costs can readily occur from changes in biomechanics of walking that occur at faster and slower frequencies than the PSF. For further research that supports this argument see Holt et al (1995, Journal of Motor Behavior) cited in the current manuscript, but also Hatsopoulos & Warren (1996, Journal of Motor Behavior), and Kuo (2001, Transactions of the ASME).
Line 388: What is the “inherent rhythm” of CPGs in human walking?

Line 391: Walking at faster or slower stride frequencies that the PSF requires greater muscle activation of the gastrocnemius and tibialis anterior, while walking at -10%PSF resulted in less activity of the rectus femoris and biceps femoris (Russell & Apatoczky, 2016 Gait & Posture). The notion of increased central drive may fit for GA and TA muscles but not RF and BF muscles. Also, it is not clear why reduced motoneuron excitability would need to be invoked.
Line 438: Note there are additional differences between PSF2 and PSF3. In PSF2, participants must listen to a monotonous metronome for 15 mins, which could change their perceptions. Secondly, they must coordinate their steps with the beat. If they touch the ground early, they will need to adjust their next step. Adjusting steps involves increased energetic cost. For treadmill walking without a metronome adjustments must be made to stay on the belt but can be made less often.
Conclusion
Line 476: Promoting the self-selection of stride frequency may be appropriate but exercise seems to contradict the notion of overloading the body to achieve changes in physiological systems.
Figures
What is NSF?
Why are the figures missing a condition?”

**C- Our Response:** We have addressed the reviewer's other specific comments as follows:

1) **Abstract:** The last part has been rephrased as suggested (page 2, lines 30-31).
2) **Introduction:** Stride Frequency was chosen because to our knowledge no previous study has examined the interplay between energy cost and affective responses when stride frequency is manipulated.
3) **Method:** We did not include the V̇O2max test protocol since V̇O2max was used solely as an inclusion criterion and was not included as an outcome variable. The protocol we used is as follows:

The incremental test includes three-minute stages (i.e., step test) starting at 2km/h and increasing by 1km/h every 3 minutes. After reaching 8 km/h, the speed was increased by 1km/h every 30 seconds until participants were exhausted. After five minutes of a recovery period, participants were asked to run at 105% of their maximal running speed achieved during the test to verify whether the true V̇O2max was completed.

4) **Method:** We have corrected the sampling rate. It was 120 Hz, not 250 Hz (page 10, line 200). Thank you for catching this error.
5) **Method:** As explained previously, the dependent samples t-tests were used specifically to compare the stride frequencies between conditions (e.g., PSF3 vs. -10%PSF2) to confirm our experimental manipulation was successful. This is a manipulation check, separate from the main 3-way mixed-design ANOVA analyses we used for the dependent variables. The use of “within conditions” made the sentence confusing so we replaced that with “of the conditions” at line 246. To clarify, we have revised the whole sentences to be more precise on page 12, lines 246-247.
6) **Method:** As explained in the response to the first comment, we used the planned contrasts resulted from the 3-way mixed-design ANOVA for our analyses because they were driven by *a priori* hypotheses. We had three specific comparisons of interest: PSF3 vs. -10%PSF2, PSF3 vs. +10%PSF2, and PSF3 vs. PSF2. As we have explained previously, PSF3 was our theoretical "optimal" reference. A post-hoc test like Bonferroni would have compared all six possible pairwise combinations, including ones with no theoretical basis (e.g., -10%PSF2 vs. +10%PSF2). This would have increased the risk of Type II error (failing to find a true effect) by correcting for multiple comparisons that were not of primary interest. Our approach of using three planned contrasts with a per-comparison alpha of 0.05 is statistically valid and more powerful for our specific, pre-planned questions. We have clarified this rationale in the manuscript. Hence, we have expanded our explanation in the statistical Analysis section, page 12, lines 251-259.
7) **Results:** We have clarified the phrasing regarding body composition. "Leaner" was imprecise. We now state that women had significantly lower body weight (page 13, line 270).
8) **Results:** We removed large and kept medium for r =.48, page 14, line 295.
9) **Discussion:** We thank the reviewer for this important clarification. We agree that central pattern generators (CPGs) are inherently flexible and can produce a range of stride frequencies, as demonstrated in both animal and human studies. Our intention was not to suggest that CPGs possess a single "hardwired" frequency that is mechanically disrupted by small deviations. Rather, we aimed to convey that the *preferred* stride frequency (PSF) emerges from an optimization process involving the dynamic interaction between CPGs, supraspinal input, sensory feedback, and biomechanical constraints. When an external metronome imposes a frequency that differs from this self-organized optimum, it requires continuous cognitive and neuromuscular adjustment—what we termed "disruption" of the natural coordination dynamics. Hence, as mentioned previously, we revised the Discussion to include a consideration of biomechanical factors on pages 19-20, lines 410-429.
10) **Discussion:** We agree that the phrase “inherent rhythm” was imprecise and potentially misleading. CPGs do not possess a single fixed rhythm; rather, they can generate a range of rhythmic outputs modulated by sensory feedback and supraspinal input. Thus the “inherent rhythm” was removed.
11) **Discussion:** We thank the reviewer for directing us to the valuable work of Russell and Apatoczky (2016). This study indeed demonstrates that muscle activation changes with stride frequency manipulation are not uniform across all lower limb muscles. While the gastrocnemius and tibialis anterior show increased activation at non-preferred frequencies, the rectus femoris and biceps femoris may show reduced activity at slower frequencies. This nuanced pattern suggests that the relationship between central drive and peripheral muscle activation is complex and muscle-specific. Regarding motoneuron excitability, we agree that invoking this mechanism is speculative based on our data. We had reframed this as a hypothesis requiring direct neurophysiological investigation, citing the work of Power et al. (2018) as a potential explanatory framework rather than a conclusion drawn from our results. Thus, after the review, we removed the paragraph detailing neuron excitability, which was on page 19, lines 394-403 in the original manuscript.
12) **Discussion:** We thank the reviewer for this insightful observation, which adds important nuance to our interpretation of the PSF2 vs. PSF3 differences. We agree that several factors beyond perceived autonomy likely contributed to the observed effects. As the reviewer notes, participants in PSF2 had to: (1) listen to a monotonous metronome for 15 minutes, which could independently influence affective state, and (2) continuously adjust their foot placement to maintain synchrony with the beat, which imposes a cumulative energetic cost (Repp & Su, 2013; Moumdjian et al., 2018). These adjustments are more frequent and demanding than the occasional corrections needed to maintain position on a treadmill during self-paced walking (PSF3).

We have expanded our discussion of the PSF2 vs. PSF3 comparison to incorporate these points on pages 22-23, lines 471-488.

13) **Conclusion:** We thank the reviewer for this important observation. We agree that there is an inherent tension between promoting self-selected intensity (to enhance adherence) and ensuring sufficient overload for physiological adaptation. Our conclusion was not intended to suggest that self-selection should replace all forms of prescribed overload, but rather that allowing individuals to self-select their stride frequency may optimize the *psychological experience* of walking, which is critical for long-term adherence (Ekkekakis et al., 2011; Williams et al., 2008).

Importantly, as Rose and Parfitt (2008) demonstrated, when individuals self-select intensity to maintain a positive affective state (e.g., feeling "good" or "fairly good"), they naturally choose intensities that fall within the ACSM-recommended range for health and fitness benefits. In our study, PSF3 elicited a pleasant affective state while still requiring measurable energy expenditure. Thus, self-selection does not preclude overload; it may simply help individuals find a sustainable intensity within the effective training zone. We thus revised the conclusion to acknowledge the overload principle and added the following sentence on page 25, lines 538-542.

14) **Figures:** we believe that there was an error in the added figure. NSF (Normal Stride Frequency) refers to the old nomenclature of the Preferred Stride Frequency (PSF). We made sure that this was corrected.

We remain available to address any future questions or edits requests by the editorial board or reviewers regarding our submission.
