# Supplementary material for: Psychophysiological responses to stride frequency manipulation in men and women
Source: PLoS One. 2026 Aug 5;21(8):e0355226. doi: 10.1371/journal.pone.0355226 (PMC13440853; doi:10.1371/journal.pone.0355226)
Supplement: Supplementary file 4 [file pone.0355226.s005.docx]

**Second Response to Reviewers**

**Second Revision**

*“Psychophysiological responses to stride frequency manipulation in men and women”*

**[ALREADY ADDRESSED]** = done in the first revision  **[NEW]** = added in this revision  **[FRAMING / SCOPE]** = respectfully clarified

Dear Dr. Ardigò,

We thank you and the reviewers for the continued, constructive engagement with our manuscript. We are encouraged that Reviewer #1 considers the manuscript improved with a single remaining request, and that Reviewer #3 now states that we responded “thoroughly and satisfactorily” to all prior feedback with no further concerns. As you requested, we have given particular attention to Reviewer #2.

In reading Reviewer #2’s list, we noted that a number of the points concern issues that were already addressed in our first revision, and we respectfully draw attention to the relevant text rather than describing those changes as new. Reviewer #2’s comments fall into three groups: (i) concerns already addressed in the previous revision; (ii) matters of theoretical framing, which we have simplified to remove ambiguity; and (iii) requests that fall outside the scope of a single revision or of PLOS ONE’s publication criteria. We address every point below, and we have made several further additions this round to strengthen the manuscript.

# **Reviewer #1**

***Comment.*** *It seems that the present version of the manuscript has been improved. Nevertheless, I still think that the ‘perceived effort (RPE)’ should be revised to ‘rating of perceived exertion (RPE)’.*

**Response. [NEW]** We thank the reviewer for prompting greater precision. We agree, following Abbiss et al. (2015), that effort and exertion are related but slightly different constructs that are often used interchangeably and are difficult to separate with a single scale and standard instructions. We have made our usage conservative and consistent throughout: the instrument is now referred to as Borg's Rating of Perceived Exertion (RPE) scale, values are reported as RPE scores, and RPE is described in its standard sense as the perceived intensity of exertion/effort during the task. We have removed any claim that the scale measures a distinct "effort" or central-drive construct, as we did not collect the concurrent perceptual or neurophysiological measures that prior work has required to demonstrate such a dissociation.

Regarding the standardized instructions noted in our Methodology: we have retained them, because instruction wording is known to influence RPE (Abbiss et al., 2015). We have, however, clarified their purpose. The leg-focused prompt ("how do you perceive the difficulty in keeping your legs active at this moment?") was used to keep the perceptual referent constant across conditions, not as a means of measuring effort separately from exertion. The interpretation that the elevated RPE under imposed pacing may reflect central/cognitive demands now appears only in the Discussion, framed as a possible mechanism consistent with the psychobiological literature and as a direction for future work.

We are grateful for this suggestion, which also resolves the related construct-definition concern raised by Reviewer #2 (point 3).

# **Reviewer #2**

We thank Reviewer #2 for the demanding review and have treated every item below. We note that the Reviewer indicated “all comments have been addressed,” and that several listed points were in fact addressed in our first revision; for those, we point to the existing text. Where a concern matches one raised by Reviewer #3 in the first round, we note that Reviewer #3 now regards it as fully resolved.

## **1. Statistical power and the sex comparison**

***Comment.*** *Sample size (n = 19; 11 vs 8) is severely underpowered for between-sex comparisons, yet sex differences remain a central framing element. Even after revision, the inferential structure still leans on absence of evidence.*

**Response. [ALREADY ADDRESSED]** Our first revision already added a post-hoc power analysis to the Limitations -with 11 men and 8 women, the minimum detectable between-group effect at 80% power and α = 0.05 is approximately Cohen’s d = 1.38, and power to detect d = 0.5 is only ~17% - and already states that the absence of significant sex differences must be read as “no difference detected,” not as equivalence (Limitations, [p. 25, lines 530-534]; Abstract , “in our sample”). **[NEW]** To remove any sense that sex remains a central claim, we have now relabelled the between-sex comparison explicitly as a **secondary, exploratory aim** (Introduction, aims paragraph, [lines 89]). Our primary hypothesis is tested within subjects, where the repeated-measures design retains adequate sensitivity; no further change to the sex comparison is possible without new data.

## **2. Precision of the central conclusion**

***Comment.*** *Claim that PSF ‘minimizes psychological responses’ is imprecise and partially contradicted by results (valence increases rather than ‘responses’ being minimized).*

**Response. [ALREADY ADDRESSED]** This wording was corrected in our first revision. The Abstract and Conclusion already state that the preferred stride frequency minimizes energy cost and RPE and is associated with higher positive valence: we no longer claim that psychological responses are “minimized” (Abstract, [p. 2, line 31]; Conclusion, [lines 547-548]). This also reflects the identical observation made by Reviewer #3 in the first round, which Reviewer #3 now considers resolved.

## **3. Construct definitions: effort vs. exertion and Borg usage**

***Comment.*** *Inconsistent distinction between ‘effort’ and ‘exertion’; operationalization of RPE deviates from standard Borg usage without sufficient validation.*

**Response. [NEW]** We thank the reviewer for prompting greater precision. We agree, following Abbiss et al. (2015), that effort and exertion are related but slightly different constructs that are often used interchangeably and are difficult to separate with a single scale and standard instructions. We have made our usage conservative and consistent throughout: the instrument is now referred to as Borg's Rating of Perceived Exertion (RPE) scale, values are reported as RPE scores, and RPE is described in its standard sense as the perceived intensity of exertion/effort during the task. We have removed any claim that the scale isolates a distinct "effort" or central-drive construct, as we did not collect the concurrent perceptual or neurophysiological measures that prior work has required to demonstrate such a dissociation.

Regarding the standardized instructions noted in our Methodology: we have retained them, because instruction wording is known to influence RPE (Abbiss et al., 2015). We have, however, clarified their purpose. The leg-focused prompt ("how do you perceive the difficulty in keeping your legs active at this moment?") was used to keep the perceptual referent constant across conditions, not as a means of measuring effort separately from exertion. The interpretation that the elevated RPE under imposed pacing may reflect central/cognitive demands now appears only in the Discussion, framed as a possible mechanism consistent with the psychobiological literature and as a direction for future work.

## **4. PSF2 vs. PSF3 — cognitive load, attentional demand, metronome effects**

***Comment.*** *The imposed vs. self-selected condition conflates stride frequency effects with cognitive load, attentional demand, and metronome-induced affective changes. This is not cleanly isolated.*

**Response. [ALREADY ADDRESSED] [FRAMING / SCOPE]** We would respectfully clarify that the PSF2-vs-PSF3 contrast is not a confound to be removed but is the comparison of interest: PSF2 and PSF3 are matched on stride frequency and walking speed, so the contrast isolates the effect of *imposing* an otherwise-preferred frequency. Our first revision already discussed the synchronization and attentional demands of metronome pacing as the leading candidate mechanism for the PSF2–PSF3 difference and noted this in the Discussion (Jäncke et al., 2000; Repp & Su, 2013; Moumdjian et al., 2018).

## **5. Statistical design: t-tests, planned contrasts, multiple comparisons, effect sizes**

***Comment.*** *Use of multiple paired t-tests risks inflated Type I error. Justification for planned contrasts is post hoc and not transparently pre-registered. Lack of correction strategy for multiple comparisons. Effect size interpretation inconsistencies.*

**Response. [ALREADY ADDRESSED] [FRAMING / SCOPE]** This concern is substantively identical to the one raised by Reviewer #3 in the first round; in the present round Reviewer #3 states that the statistical analysis has been addressed “thoroughly and satisfactorily” with “no further concerns.” For clarity: the primary inferential model is a repeated-measures (mixed) ANOVA, not a series of paired t-tests; paired t-tests appear only as a manipulation check on stride frequency, as already stated in the Methodology. The planned contrasts test an *a priori* set of theory-driven comparisons (PSF3 vs +10%PSF2; PSF3 vs −10%PSF2; PSF3 vs PSF2), and the rationale for choosing focused contrasts over omnibus post-hoc tests is already given in the first revision. PLOS ONE does not require pre-registration. **[NEW]** To address the multiple-comparison point directly, we have added a **sensitivity analysis** applying a Benjamini-Hochberg false-discovery-rate (FDR) correction to the family of planned contrasts within each primary outcome (Supporting Information, Table S1). After correction, the condition effects on V̇O₂ and on RPE remained significant for all contrasts (adjusted *p* ≤ .04), and the Feeling Scale contrasts remained significant at the conventional threshold (adjusted *p* ≈ .05). We note in the Results and Discussion that the valence effect, while surviving correction, is of more modest robustness than the metabolic and perceptual effects, and we have calibrated the valence wording accordingly.

## **6. Measurement validity: ordinal scales, time-averaging, inter-rater reliability**

***Comment.*** *Ordinal affective scales treated with parametric summaries without justification. Averaging across time (3–15 min) obscures temporal dynamics. No reliability reporting for dual-observer stride counts.*

**Response. [NEW]** (i) We agree this warranted explicit justification. We have added a statement to the Statistical Analysis section noting that, although the Feeling Scale, Felt Arousal Scale, and RPE yield ordinal ratings, parametric analysis follows established practice for affective and psychophysical scales in exercise science (Hardy & Rejeski, 1989; Ekkekakis et al., 2011) and is supported by evidence that parametric tests are robust to multi-category ordinal data (Norman, 2010). We also acknowledge the ordinal nature of these measures as a consideration in the Limitations. **[ALREADY ADDRESSED]** (ii) Temporal dynamics: time was already modelled as a within-subject factor in the ANOVA; we report the significant main effect of time on RPE (F (1.524, 25.901) = 16.349, *p* < 0.001) and the per-time-point values, so the time course is not lost. **[NEW]** (iii) Inter-rater reliability: stride counts were performed by two researchers who counted independently and blind to one another, and the mean of the two counts was used for each window specifically to minimize single-observer error. We have made this procedure explicit in the Methodology, lines 215-217.

## **7. Instrumentation: camera sampling rate**

***Comment.*** *Reported camera sampling rate error (250 Hz vs 120 Hz) indicates poor methodological control.*

**Response. [ALREADY ADDRESSED]** This was corrected in our first revision. The Methods already state that the Sony ZV-1M2 was operated at **a sampling rate of 120 Hz** (p. 10, line 204). the earlier 250 Hz value has not appeared since the original submission. We confirm that all stride-counting windows and analyses used this verified 120 Hz rate, and that the configuration does not affect any reported result.

## **8. Control of confounders: menstrual phase, monotony, learning effects**

***Comment.*** *Menstrual cycle phase not biologically verified. Psychological state influenced by metronome monotony not controlled. No control for learning or adaptation effects across conditions.*

**Response. [ALREADY ADDRESSED] [FRAMING / SCOPE]** (i) Menstrual phase: our first revision already states that we scheduled female participants in the follicular/non-active phase using a Menstrual Cycle Survey and a validated backward-count method (Schmalenberger et al., 2020), and already acknowledges in the Limitations that hormonal assays were not used and that some phase misclassification is possible (lines 547-550). Hormonal verification was beyond the scope and budget of this study; because the sex comparison is secondary, residual phase variability does not affect our primary within-subject conclusions. (ii) Metronome monotony: addressed under point 4 as part of the mechanism under study. (iii) Learning/adaptation: each condition was preceded by familiarization, sessions were separated by a 48-hour washout, and the four conditions were counterbalanced, as stated in the Methodology

## **9. Mechanistic interpretation (central command, CPG, neuromotor)**

***Comment.*** *Claims about central command, CPG disruption, and neuromotor processes lack direct measurement. Neurophysiological explanations exceed evidential basis.*

**Response. [ALREADY ADDRESSED]** We agree we did not measure neural activity directly. Our first revision already softened these passages with the consistent use of “may”, “likely” and “perhaps”. We retain them only as theoretically grounded interpretation.

## **10. Biomechanical determinants**

***Comment.*** *Insufficient integration of established biomechanical determinants of stride efficiency.*

**Response. [ALREADY ADDRESSED]** Our first revision already added a biomechanical discussion, presented alongside the neural account as a parallel, non-exclusive explanation for the increased cost and effort at non-preferred frequencies, drawing on the literature recommended in the first round (Holt et al., 1995; Hatsopoulos & Warren, 1996; Kuo, 2001; Russell & Apatoczky, 2016). The Reviewer notes this is “partially corrected”.

## **11. External validity and behavioral extrapolation**

***Comment.*** *Homogeneous sample (young, healthy, active) undermines generalizability. Overextension to exercise adherence and broader behavioral claims.*

**Response. [ALREADY ADDRESSED]** Our first revision already states in the Limitations that the findings generalize only to young, healthy, physically active adults, and in the Conclusion, we have already rephrased the adherence and behavioral statements as clearly labelled implications and directions for future research rather than demonstrated outcomes.

## **12. Data availability**

***Comment.*** *Restricted dataset access may conflict with journal policy despite mitigation.*

**Response. [ALREADY ADDRESSED]** We note that in the present round all three reviewers, including Reviewer #2, answered “Yes” to whether the data underlying the findings have been made fully available. As stated in our Data Availability Statement, the underlying dataset has been deposited at Mendeley Data under restricted access to protect participant anonymity, as mandated by our ethics approval; qualifying researchers may apply for access through the Health Research Ethics Board (T: +1 709 864 8871) subject to a Data Use Agreement. This arrangement follows the provisions of the PLOS Data policy for data subject to ethical restrictions, and we believe the concern is resolved.

## **13. Clarity and reporting (condition labels, figures, language)**

***Comment.*** *Terminological inconsistency (PSF1, PSF2, PSF3 logic not intuitively clear). Figures and labels (e.g., NSF) previously inconsistent. Some grammatical and phrasing errors persist.*

**Response. [ALREADY ADDRESSED]** The erroneous “NSF” label issue in the figures was corrected following the first round.

## **14. Theoretical contribution and novelty**

***Comment.*** *Largely confirmatory of known U-shaped energy cost relationships. Incremental contribution framed as broader psychophysiological insight without sufficient novelty.*

**Response. [FRAMING / SCOPE]** We respectfully note that PLOS ONE’s stated criteria base editorial decisions on scientific and methodological soundness rather than on perceived novelty or impact (PLOS ONE, Criteria for Publication; Editorial and Peer Review Process). We have accordingly directed our revisions toward rigour, transparency, and appropriately calibrated conclusions. We would add that, to our knowledge, this is the first study to characterize metabolic, perceptual, and affective responses jointly across imposed versus self-selected stride frequencies matched on frequency and speed (the PSF2-vs-PSF3 contrast). We have stated this specific contribution in the Introduction without overstating its significance, consistent with the journal’s guidance.

In summary, the majority of Reviewer #2’s points were addressed in our first revision, to which we have drawn attention above; the remaining points have been resolved in this revision through the simplified RPE terminology, the added Benjamini-Hochberg sensitivity analysis confirming the robustness of the primary outcomes, the clarified inter-rater procedure, the more conservative framing of the secondary sex comparison and the behavioral implications. We are grateful to all three reviewers and to the Academic Editor, and we hope the manuscript is now suitable for publication. We would, of course, welcome any further guidance.

Sincerely,

The Authors
