# Supplementary material for: Psychophysiological responses to stride frequency manipulation in men and women
Source: PLoS One. 2026 Aug 5;21(8):e0355226. doi: 10.1371/journal.pone.0355226 (PMC13440853; doi:10.1371/journal.pone.0355226)
Supplement: Supplementary file 5 [file pone.0355226.s006.docx]

**Response to Reviewers**

**Manuscript PONE-D-25-68663R2**

*“Psychophysiological responses to stride frequency manipulation in men and women”*

We thank the Editor and Reviewers for their continued engagement with our manuscript. We are grateful that both Reviewer #1 and Reviewer #2 indicate that their comments have been satisfactorily addressed, and that Reviewer #2 confirms the manuscript is technically sound with appropriate and rigorous statistical analyses. We respond to the remaining points raised by the reviewers below.

**Reviewer #1**

We thank Reviewer #1 for the positive assessment. Regarding the Data Availability Statement, the dataset has already been deposited in Mendeley Data under restricted access to protect participant anonymity, in accordance with our approved ethics protocol. Qualified researchers may request access through the Health Research Ethics Board (Tel: +1 709 864 8871; www.hrea.ca), subject to ethical and governance requirements.

**Reviewer #2**

We are grateful that Reviewer #2 now regards the manuscript as technically sound (Q2: Yes) and the statistical analysis as appropriate and rigorous (Q3: Yes), and further indicates that all comments have been satisfactorily addressed. After carefully considering the accompanying remarks, we find that they converge on a single overarching theme: the need to clearly distinguish observation from interpretation and to ensure that mechanistic and behavioural inferences are framed with appropriate caution. We fully agree with this principle and respectfully note that it is already reflected throughout the revised manuscript. Rather than responding to each comment individually, we highlight below where the substantive points raised by the reviewer are addressed in the current text:

**Interpretation presented as hypothesis rather than conclusion.** The Discussion already frames the proposed neural and central-command mechanisms as hypotheses rather than definitive explanations. The text explicitly acknowledges that these mechanisms are “not mutually exclusive” with biomechanical explanations, employs appropriately cautious language throughout (e.g., “we postulate,” “may,” “perhaps,” and “likely”), and notes—specifically with respect to the proposed central origin of perceived exertion—that this interpretation is made “although we did not measure this directly.” Accordingly, no neurophysiological mechanisms are presented as observed findings; rather, they are discussed as plausible interpretations of the results that remain to be tested in future research.

**PSF2 versus PSF3.** The manuscript already addresses the possibility that metronome pacing may impose additional attentional and synchronization demands. Specifically, the Discussion identifies the “cognitive and neuromuscular demands of auditory–motor synchronization” as a potential contributing factor and interprets the PSF2 effect in relation to perceived autonomy and cognitive appraisal. This distinction between the mechanisms potentially underlying PSF2 and PSF3 was clarified in response to comments from other reviewers during earlier review rounds and was considered satisfactorily resolved. We have explained this contrast on several occasions throughout the revision process and believe that it is now clearly articulated in the manuscript.

**Sex comparison.** The between-sex analysis is already clearly identified as a secondary objective. The manuscript explicitly acknowledges that this analysis is underpowered, provides the corresponding power calculation (minimum detectable effect size *d* ≈ 1.38; approximately 17% power to detect a medium effect), and states that the findings should be interpreted “not as evidence of equivalence.” Throughout the manuscript, sex-related results are reported cautiously and exclusively in terms of an absence of statistically detected differences, without implying that males and females respond similarly or that meaningful differences do not exist. This interpretation is therefore consistent with the limitations of the available sample size and statistical power.

**Ordinal scales and statistical analyses.** The manuscript already provides a clear justification for the use of parametric analyses with these measures, drawing on established practice in the affective and exercise science literature (Hardy & Rejeski, 1989; Ekkekakis et al., 2011) as well as evidence regarding the robustness of parametric methods applied to ordinal data (Norman, 2010). In addition, a Benjamini–Hochberg sensitivity analysis is reported in Supplementary Table S1, and the ordinal nature of the measures is explicitly acknowledged as a limitation. We also note that Reviewer #2 has now assessed the statistical analyses as appropriate and rigorous. Accordingly, this issue is already addressed in the current manuscript through methodological justification, sensitivity analyses, and a transparent discussion of limitations.

**Novelty.** We retain a single, accurate statement of the manuscript’s contribution: the joint characterization of metabolic, perceptual, and affective responses across imposed and self-selected stride frequency conditions matched for both stride frequency and walking speed. We respectfully note that *PLOS ONE* evaluates manuscripts primarily on the basis of scientific and methodological soundness rather than perceived novelty or impact. The manuscript therefore makes no claims beyond its specific contribution and is presented in a manner consistent with the journal’s publication criteria.

For these reasons, we have not introduced further modifications to the manuscript. We believe that the substantive points raised by Reviewer #2 are already incorporated into the current manuscript and that further modification of the Discussion would add little to the interpretation already presented. Moreover, additional revisions risk compromising the concision, balance, and focus of the manuscript, which have been positively assessed by Reviewers #1 and #3. We are grateful to Reviewer #2 for the rigor and careful consideration they have brought to the review process, which has helped strengthen the manuscript. We therefore respectfully seek the Academic Editor’s guidance on the most appropriate way forward.
