# Supplementary material for: Psychophysiological responses to stride frequency manipulation in men and women
Source: PLoS One. 2026 Aug 5;21(8):e0355226. doi: 10.1371/journal.pone.0355226 (PMC13440853; doi:10.1371/journal.pone.0355226)
Supplement: Peer review history

# Peer review: Decision Letter 0

**DOI:** 10.1371/journal.pone.0355226.r001

**Authors:** Luca Ardigò

18 Feb 2026

PONE-D-25-68663

Psychophysiological responses to stride frequency manipulation in men and women

PLOS One

Dear Dr. Basset,

Thank you for submitting your manuscript to PLOS ONE. After careful consideration, we feel that it has merit but does not fully meet PLOS ONE’s publication criteria as it currently stands. Therefore, we invite you to submit a revised version of the manuscript that addresses the points raised during the review process.

Please submit your revised manuscript by Apr 04 2026 11:59PM. If you will need more time than this to complete your revisions, please reply to this message or contact the journal office at plosone@plos.org. When you're ready to submit your revision, log on to https://www.editorialmanager.com/pone/ and select the 'Submissions Needing Revision' folder to locate your manuscript file.

Please include the following items when submitting your revised manuscript:

- A letter that responds to each point raised by the academic editor and reviewer(s). You should upload this letter as a separate file labeled 'Response to Reviewers'.
- A marked-up copy of your manuscript that highlights changes made to the original version. You should upload this as a separate file labeled 'Revised Manuscript with Track Changes'.
- An unmarked version of your revised paper without tracked changes. You should upload this as a separate file labeled 'Manuscript'.

If you would like to make changes to your financial disclosure, please include your updated statement in your cover letter. Guidelines for resubmitting your figure files are available below the reviewer comments at the end of this letter.

If applicable, we recommend that you deposit your laboratory protocols in protocols.io to enhance the reproducibility of your results. Protocols.io assigns your protocol its own identifier (DOI) so that it can be cited independently in the future. For instructions see: https://journals.plos.org/plosone/s/submission-guidelines#loc-laboratory-protocols. Additionally, PLOS ONE offers an option for publishing peer-reviewed Lab Protocol articles, which describe protocols hosted on protocols.io. Read more information on sharing protocols at https://plos.org/protocols?utm_medium=editorial-email&utm_source=authorletters&utm_campaign=protocols.

We look forward to receiving your revised manuscript.

Kind regards,

Luca Paolo Ardigò, Ph.D.

Academic Editor

PLOS One

Journal Requirements:

When submitting your revision, we need you to address these additional requirements.

1. Please ensure that your manuscript meets PLOS ONE's style requirements, including those for file naming. The PLOS ONE style templates can be found at

https://journals.plos.org/plosone/s/file?id=wjVg/PLOSOne_formatting_sample_main_body.pdf and

https://journals.plos.org/plosone/s/file?id=ba62/PLOSOne_formatting_sample_title_authors_affiliations.pdf

2.  We note that you have indicated that there are restrictions to data sharing for this study. For studies involving human research participant data or other sensitive data, we encourage authors to share de-identified or anonymized data. However, when data cannot be publicly shared for ethical reasons, we allow authors to make their data sets available upon request. For information on unacceptable data access restrictions, please see http://journals.plos.org/plosone/s/data-availability#loc-unacceptable-data-access-restrictions.

Before we proceed with your manuscript, please address the following prompts:

a) If there are ethical or legal restrictions on sharing a de-identified data set, please explain them in detail (e.g., data contain potentially identifying or sensitive patient information, data are owned by a third-party organization, etc.) and who has imposed them (e.g., a Research Ethics Committee or Institutional Review Board, etc.). Please also provide contact information for a data access committee, ethics committee, or other institutional body to which data requests may be sent.

b) If there are no restrictions, please upload the minimal anonymized data set necessary to replicate your study findings to a stable, public repository and provide us with the relevant URLs, DOIs, or accession numbers. Please see http://www.bmj.com/content/340/bmj.c181.long for guidelines on how to de-identify and prepare clinical data for publication. For a list of recommended repositories, please see https://journals.plos.org/plosone/s/recommended-repositories. You also have the option of uploading the data as Supporting Information files, but we would recommend depositing data directly to a data repository if possible.

Please update your Data Availability statement in the submission form accordingly.

3. If the reviewer comments include a recommendation to cite specific previously published works, please review and evaluate these publications to determine whether they are relevant and should be cited. There is no requirement to cite these works unless the editor has indicated otherwise.

**Additional Editor Comments:**

Please, address point-to-point all issues raised by the three expert reviewers.

[Note: HTML markup is below. Please do not edit.]

Reviewers' comments:

Reviewer's Responses to Questions

**Comments to the Author**

1. Is the manuscript technically sound, and do the data support the conclusions?

The manuscript must describe a technically sound piece of scientific research with data that supports the conclusions. Experiments must have been conducted rigorously, with appropriate controls, replication, and sample sizes. The conclusions must be drawn appropriately based on the data presented.

Reviewer #1: Partly

Reviewer #2: Yes

Reviewer #3: Yes

**********

2. Has the statistical analysis been performed appropriately and rigorously?

Reviewer #1: I Don't Know

Reviewer #2: No

Reviewer #3: No

**********

3. Have the authors made all data underlying the findings in their manuscript fully available?

The PLOS Data policy requires authors to make all data underlying the findings described in their manuscript fully available without restriction, with rare exception (please refer to the Data Availability Statement in the manuscript PDF file). The data should be provided as part of the manuscript or its supporting information, or deposited to a public repository. For example, in addition to summary statistics, the data points behind means, medians and variance measures should be available. If there are restrictions on publicly sharing data—e.g. participant privacy or use of data from a third party—those must be specified.

Reviewer #1: No

Reviewer #2: No

Reviewer #3: No

**********

4. Is the manuscript presented in an intelligible fashion and written in standard English?

PLOS ONE does not copyedit accepted manuscripts, so the language in submitted articles must be clear, correct, and unambiguous. Any typographical or grammatical errors should be corrected at revision, so please note any specific errors here.

Reviewer #1: Yes

Reviewer #2: Yes

Reviewer #3: Yes

**********

5. Review Comments to the Author

Please use the space provided to explain your answers to the questions above. You may also include additional comments for the author, including concerns about dual publication, research ethics, or publication ethics. (Please upload your review as an attachment if it exceeds 20,000 characters)

Reviewer #1: Overview

This study compared metabolic cost and perceptual responses during walking at preferred stride frequency (PSF), PSF+10%, and PSF-10% conditions in men and women. It was reported that PSF condition elicited lower oxygen uptake, lower rating of perceived exertion, and higher positive valence compared to the ±10% deviations. The authors have concluded that PSF minimizes energy cost and psychological responses during walking, with no difference between men and women.

General comments

Although research on perceptual responses during exercise seems interesting, there are detailed questions/suggestions listed below that should be considered. For example, it is not clear why PSF3 condition was used as a landmark condition to identify the influence of stride frequency on the measured variables. I think that the following comparisons may be useful for this study: PSF2 versus PSF2+10%, PSF2 versus PSF2-10%, and PSF2 versus PSF3. Furthermore, I think that oxygen uptake can be normalized to lean body mass in this study.

Specific Comments

1. Title

1) I think that the title of the article can be revised for more clarity. For example, I think that the ‘Psychophysiological responses’ can be changed to ‘Metabolic cost and perceptual responses’.

2. Abstract

1) I think that the ‘psychophysiological responses’ can be changed (e.g., metabolic cost and perceptual responses).

2) I think that the ‘perceived effort (RPE)’ should be revised to ‘rating of perceived exertion (RPE)’.

3. Introduction

1) I think that ‘psychological responses’ can be changed (e.g., perceptual responses).

2) I think that more detailed information regarding the ‘central pattern generators’ can be provided.

4. Methodology

1) What is the scientific rationale for the determination of the duration (i.e., 15 min)?

2) I think that the ‘Rating of Perceived Effort’ can be changed to ‘Rating of Perceived Exertion’.

5. Results

1) Please double-check the accuracy of the statistical methods (e.g., The dependent samples t-test (Page 13, Lines 260~265)).

2) It is not clear why PSF3 (not PSF2) condition was used to identify the influence of stride frequency on the measured variables.

6. Discussion

1) I think that most of the Discussion section is based upon speculations. I think that the article would be stronger if the authors could focus more on their research outcomes (e.g., relatively small difference in rating of perceived exertion). Furthermore, I think that the following article may possibly be useful for the interpretation/discussion regarding oxygen uptake and perceptual responses:

・Rose and Parfitt. Medicine and Science in Sports and Exercise, 40(10), 1852–1860, 2008.

2) I think that it may be interesting to present each participant’s felt arousal scale data.

3) I think that the information regarding the central pattern generators (Page 18, Lines 379~386) can be moved to the Introduction section.

4) I think that the ‘small sample size’ may not be related to the ‘generalizability’ (Page 22, Lines 460~461).

5) I think that this sentence (Page 22, Lines 473~475) can be revised (e.g., ‘small’)

7. Figures

1) I think that the y-axis of the Figures can be corrected (e.g., I think that rating of perceived exertion can be presented from 6 to 20).

Reviewer #2: Thank you for submitting your manuscript. While the study investigates an interesting aspect of gait psychophysiology and employs a rigorous control for the menstrual cycle, I must recommend rejection based on several fundamental methodological and interpretive flaws.

1. Unsupported Primary Conclusions (Statistical Power)

A central conclusion of the manuscript is that there are "no differences between men and women". However, with a sample size of only 8 women and 11 men, the study is severely underpowered for between-subjects comparisons. The failure to detect a significant difference is highly likely a Type II error rather than proof of equivalence. Basing a primary conclusion on an underpowered null finding is a fatal flaw in the analysis.

2. Extreme Over-Interpretation of Mechanisms

The discussion relies heavily on speculative mechanisms to explain the differences between the PSF2 and PSF3 conditions. Attributing the increased perceived effort and decreased pleasure to "diminished perceived autonomy," "central command," and "increased brain metabolic demand" is entirely unsubstantiated by the data. The study includes no neurophysiological (e.g., EEG, fMRI) or psychological autonomy measures to support these claims, making the discussion highly conjectural rather than data-driven.

3. Violation of Data Availability Policy

The manuscript currently violates PLOS ONE’s strict Open Data policy. Stating that data is only available "from the corresponding author upon reasonable request" due to ethics restrictions is not acceptable. For a manuscript to be considered, authors must either provide the minimal anonymized dataset or provide the direct contact information of an ethics committee or legal body that fields data requests.

4. Limited Generalizability

The small sample size (N=19) of strictly young, healthy, and physically active individuals severely limits the generalizability of the findings, particularly given the broad claims made about human locomotion and exercise adherence.Given the fundamental issues with statistical power and the speculative nature of the discussion, the manuscript is not suitable for publication in its current form.

Reviewer #3: PLOS One Psychophysiological responses to stride frequency manipulation in men and women

PONE-D-25-68663

Previous research has shown that oxygen consumption is minimal when walking at the preferred stride frequency (PSF) compared with faster or slower frequencies, when walking speed is maintained across conditions. This study extends that work by also assessing psychological responses to walking at different stride frequencies. This manuscript presents original research and for the most part meets the review requirements for PLOS One. I have two major concerns about statictics and interpretation of findings, and a number of smaller concerns.

Major Concerns

1. I am concerned about type I error rate in the study. Multiple paired t-tests are used to compare stride frequencies without a clear rationale for not using ANOVA. Also planned comparisons are used to compare all combinations of conditions when post hocs would be appropriate. Planned comparisons should be reserved for specific hypotheses made in the paper and should not compare all combinations.

2. The findings are interpreted in terms of changes to central command and motoneuron excitability, however, no consideration has been made for the biomechanical changes that occur with walking at different stride frequencies. There is extensive literature that considers how the biomechanics of the body impact PSF (see references below for examples). I would at least expect the authors to consider alternative explanations although of course use their results to support their claims.

Abstract

The conclusion that PSF minimizes psychological responses is not accurate. The results show it resulted in higher positive valence.

Introduction

Line 44: Why is altering stride frequency not a change in exercise intensity? Don’t the findings of previous research show that walking at non-PSF increases intensity as indicated by oxygen consumption, heart rate (HR), and rating of perceived exertion (RPE)?

Method

Line 94: When and what protocol was used to determine VO2max?

Line 194: According to a quick online search, the Sony ZV-1M2 has a maximum video frame rate of 120 Hz. Please check and confirm what was used in the experiment.

Line 239: Why were paired samples t-test used to assess stride frequency? It’s not clear why 3-way mixed design ANOVA was not used. Repeated uses of paired samples t-tests increase type I error rate. Also, be specifying “within conditions” I was expecting just comparisons within conditions but the results section seems to included comparisons between conditions.

Line 244: Why were planned contrasts used instead of post hoc tests? Planned comparisons inflate the experiment-wise type I error rate and are therefore used to address specific hypotheses and should not be used to “decompose any significant interactions or differences”. Post hoc tests are designed to test all combinations of conditions and can control experiment-wise error rate, depending on type used.

Results

Line 255: The claim that women were significantly leaner does not match the Table 1. According to the table values the two gender groups fit firmly within each other’s 95% confidence intervals.

Line 281: Specify, the interpretation of effect size magnitude in the method section. I believe Cohen would conclude that r=.48 is medium (<.5).

Discussion

Line 377: It is not clear that changing stride frequency by 10% disrupts CPGs and the locomotor control system. My understanding of central pattern generators (CPGs) is that they can readily produce a range of frequencies. What evidence is there that CPGs have a specific frequency that matches the preferred stride frequency of walking? Increased physical and perceptual costs can readily occur from changes in biomechanics of walking that occur at faster and slower frequencies than the PSF. For further research that supports this argument see Holt et al (1995, Journal of Motor Behavior) cited in the current manuscript, but also Hatsopoulos & Warren (1996, Journal of Motor Behavior), and Kuo (2001, Transactions of the ASME).

Line 388: What is the “inherent rhythm” of CPGs in human walking?

Line 391: Walking at faster or slower stride frequencies that the PSF requires greater muscle activation of the gastrocnemius and tibialis anterior, while walking at -10%PSF resulted in less activity of the rectus femoris and biceps femoris (Russell & Apatoczky, 2016 Gait & Posture). The notion of increased central drive may fit for GA and TA muscles but not RF and BF muscles. Also, it is not clear why reduced motoneuron excitability would need to be invoked.

Line 438: Note there are additional differences between PSF2 and PSF3. In PSF2, participants must listen to a monotonous metronome for 15 mins, which could change their perceptions. Secondly, they must coordinate their steps with the beat. If they touch the ground early, they will need to adjust their next step. Adjusting steps involves increased energetic cost. For treadmill walking without a metronome adjustments must be made to stay on the belt but can be made less often.

Conclusion

Line 476: Promoting the self-selection of stride frequency may be appropriate but exercise seems to contradict the notion of overloading the body to achieve changes in physiological systems.

Figures

What is NSF?

Why are the figures missing a condition?

**********

6. PLOS authors have the option to publish the peer review history of their article (what does this mean?). If published, this will include your full peer review and any attached files.

If you choose “no”, your identity will remain anonymous but your review may still be made public.

**Do you want your identity to be public for this peer review?**  For information about this choice, including consent withdrawal, please see our Privacy Policy.

Reviewer #1: No

Reviewer #2: **Yes:**  Åke Elden

Reviewer #3: No

**********

[NOTE: If reviewer comments were submitted as an attachment file, they will be attached to this email and accessible via the submission site. Please log into your account, locate the manuscript record, and check for the action link "View Attachments". If this link does not appear, there are no attachment files.]

To ensure your figures meet our technical requirements, please review our figure guidelines: https://journals.plos.org/plosone/s/figures

You may also use PLOS’s free figure tool, NAAS, to help you prepare publication quality figures: https://journals.plos.org/plosone/s/figures#loc-tools-for-figure-preparation.

NAAS will assess whether your figures meet our technical requirements by comparing each figure against our figure specifications.

Attachment Submitted filename: Major Comments_review PLOS.docx

# Author comment: Author response to Decision Letter 1

**DOI:** 10.1371/journal.pone.0355226.r002

14 Apr 2026

We thank the Academic Editor and the three reviewers for their careful reading and constructive suggestions which improved the manuscript substantially. Below we respond point-by-point to each comment and describe the changes made in the revised manuscript. Page/line numbers below refer to the tracked-changes manuscript.

Response to Journal Requirements and Editor's Comments

Journal Requirement 1 (PLOS ONE Style): We have thoroughly reviewed the PLOS ONE formatting guidelines and have ensured that our manuscript, including file naming, adheres to the required style.

Journal Requirement 2 (Data Availability): The study protocol has been approved by the Health Research Ethics Board, T: +1 709 864 8871, F: 709 864 8870, www.hrea.ca.

To comply with PLOS ONE’s data policy while adhering to the ethical constraints regarding participant privacy, the underlying data set has been deposited at Borealis Memorial University Dataverse. To protect participant anonymity as mandated by our ethics approval, the data are held under restricted access. Interested researchers who meet the criteria for access to confidential data may apply for access through [https://doi.org/10.5683/SP3/2ANOJ3], subject to approval by the Health Research Ethics Board and the signing of a Data Use Agreement.

We remain available to address any further questions the editorial board or reviewers may have regarding our submission.

Major Comments & Questions

1) Data availability compliance: answered in the previous comment.

2) Statistical Power and Sex Differences: That is a fair and critical question. We answered this also in the response to Reviewer #2.

We fully acknowledge this critical limitation. Our conclusion of "no differences between men and women" was stated too strongly given our sample size. We have revised the language throughout the manuscript to reflect that we simply did not detect a significant difference in this sample, rather than asserting equivalence. Regarding a post-hoc power analysis, we have carefully considered this point. Methodological literature strongly advises against traditional post-hoc power calculations (Hoenig & Heisey, 2001) as they are mathematically redundant with the p-value and can be misleading for data interpretation. As a more informative alternative, we conducted a post-hoc sensitivity analysis. With n=11 (men) and n=8 (women), the minimal detectable Cohen's d (80% power, two-sided α = 0.05) is ≈ 1.38, and the power to detect a medium effect (d = 0.5) is ≈ 17%. These values quantify the limited sensitivity of our between-sex comparisons.

Actions taken:

a) Abstract: it has been revised (Page 2, Line 31): “...with no significant differences detected between men and women in our sample.”

b) Discussion: it has been revised and the following was added in page 24, lines 514-519: “Second, we fully acknowledge that between-sex comparisons in this study are underpowered. With 11 men and 8 women, the minimal detectable between-group effect (Cohen's d) with 80% power and α = 0.05 is approximately 1.38; the power to detect a medium effect (d = 0.5) is only about 17%. Thus, the absence of statistically significant sex differences in our sample should be interpreted cautiously and not as evidence of equivalence. Future studies with larger and balanced samples are required to test sex differences reliably”.

c) Made clear in the “Statistical Analysis” page 12, lines 251-259, that the study's primary tests were the within-subject planned contrasts, which are appropriately powered and form the basis of our main conclusions.

We believe these revisions appropriately contextualize the null sex findings while maintaining scientific rigor.

3) Mechanistic Speculations (Central Command & Autonomy): This is an excellent point. We responded also to the same comment provided by Reviewer #2.

We agree that our initial discussion framed these mechanisms too definitively. We have now extensively revised the Discussion to frame these explanations as plausible hypotheses derived from the literature, not as conclusions directly proven by our data. We have also added a consideration of alternative biomechanical explanations (as suggested by Reviewer #3).

Actions taken:

a) Elaborated our interpretation of the RPE and FS findings. Thus, we added two paragraphs: a paragraph (page 20-21, lines 433-447) elaborating on the small difference in RPE and the relationship between RPE and pleasure, and another paragraph (page 22-23, lines 473-488) elaborating on the dissociation between PSF2 and PSF3.

b) Toned down speculative language: we used “may be attributed to” (line 493) and “perhaps” (line 494).

c) We have also added a consideration of alternative biomechanical explanations (as suggested by Reviewer #3) on pages 19-20, lines 410-429.

d) Removed the paragraph detailing neuron excitability, which was on page 19, lines 394-403 in the original manuscript.

4) Familiarization Protocol Adequacy: We thank the reviewer for this important methodological question. Our rationale for considering this sufficient was based on three factors:

a) Simplicity of the task: Synchronizing footsteps with an isochronous metronome at a self-selected walking speed is a relatively low-complexity motor task. Research on sensorimotor synchronization indicates that the basic ability to match steps to a beat is readily accessible to healthy adults, even without extensive practice (Repp & Su, 2013).

b) Prior exposure during PSF1 determination: Before the familiarization period with the metronome, participants had already walked for 15 minutes at their preferred speed while their natural stride frequency was measured. Thus, by the time they were exposed to the metronome, they were already accustomed to the treadmill and the walking speed.

c) Pilot testing: In pilot work preceding this study, we observed that pilot participants who have never been exposed to a metronome before readily matched the metronome frequency and could achieve basic synchronization within 2–3 minutes. The 5-minute period was therefore chosen to provide ample time for all participants to become comfortable.

However, we fully agree with the reviewer that task novelty cannot be entirely ruled out as a contributing factor to the elevated RPE in the metronome conditions.

Minor Comments & Clarifications

1) Figure Error Bars: We thank the reviewer for noting this. The error bars in Figures 2, 3, and 4 represent Standard Deviation (SD). We have updated the figure captions accordingly to specify this.

2) Statistical Precision: We thank the reviewer for this attention to detail. We have revised Table 1 to report all continuous variables (including BMI) with integers. We decided to use integers and not one decimal place for scientific accuracy for example for age and BMI as someone cannot be for instance 23.4 years old.

3) Language and Phrasing: We thank the reviewer for catching this grammatical error. The correct sentence is “interaction with participants and cell phone usage was refrained”. Correction was made.

4) Menstrual Cycle Verification: We thank the reviewer for this important question regarding menstrual cycle verification. We acknowledge that precise phase confirmation ideally requires hormonal assays, and the absence of such assays is a limitation of our study. We added this limitation in pages 24-25, lines 527-530.

However, we implemented several methodological controls to maximize the accuracy of phase assignment within the constraints of our study design:

a) Screening for cycle regularity: Only women with self-reported regular menstrual cycles (21–32 days) were included. Those with irregular cycles, polycystic ovary syndrome, endometriosis, or other conditions affecting cycle regularity were excluded.

b) Verification methods:

- For women who tracked their cycle using apps or calendars (n = 5), we used their tracking data to confirm follicular phase timing.

- For those who did not track (n = 3), we used the backward count method based on the projected start date of their next menses, as recommended by Schmalenberger et al. (2020) for situations where hormonal assays are not feasible.

c) Scheduling window: All sessions were scheduled within days 1–7 of the follicular phase (or the inactive phase for oral contraceptive users), which is the most reliable window for identifying early follicular phase without hormonal confirmation.

d) Confirmation at session start: At the beginning of each session, participants were asked to confirm they were menstruating or in the expected phase based on their tracking or backward count.

We remain available to address any future questions or edits requested by the editorial board or reviewers regarding our submission.

Additional Editor Comments: We have provided a point-by-point response to each of the three expert reviewers below.

We remain available to address any future questions or edits requested by the editorial board or reviewers regarding our submission.

Response to Reviewers' Comments

Reviewer #1

1- General Comment: “it is not clear why PSF3 condition was used as a landmark condition to identify the influence of stride frequency on the measured variables. I think that the following comparisons may be useful for this study: PSF2 versus PSF2+10%, PSF2 versus PSF2-10%, and PSF2 versus PSF3. Furthermore, I think that oxygen uptake can be normalized to lean body mass in this study.”

1- Our Response: we thank the reviewer for this important observation, as it highlights a critical aspect of our experimental design that we have now clarified in the manuscript. PSF3 is the true, unconstrained preferred stride frequency. The rationale for using PSF3 as the reference is that it represents a completely self-selected, natural motor pattern, free from any external constraint.

In contrast, PSF2, while numerically identical to PSF1, is an imposed condition where participants must consciously synchronize their steps with a metronome. This introduces a cognitive load that is absent during natural walking. Therefore, comparing the imposed conditions (-10%PSF2, +10%PSF2, PSF2) against the truly natural condition (PSF3) allows us to isolate the specific effect of an externally imposed constraint from the effect of the stride frequency value itself. We got inspired by Vazou-Ekkekakis & Ekkekakis, 2009 [study mentioned in the discussion section, Vazou-Ekkekakis, S., & Ekkekakis, P. (2009). Affective consequences of imposing the intensity of physical activity: does the loss of perceived autonomy matter? Hellenic Journal of Psychology, 6, 125–144.] that reported that imposed self-selected exercise intensity, as opposed to self-selected intensity, reduces pleasure.

Thus, because that contrast (PSF3 vs PSF2 and PSF3 vs ±10%PSF2) was pre-specified, we used planned contrasts (resulted from the 3-way mixed design ANOVA) with PSF3 as the reference.

Action taken: we have added a clearer explanation on page 8, lines 147-149 and page 12-13, lines 251-259.

Furthermore, regarding oxygen uptake being normalized to lean body mass, we believe that normalizing oxygen uptake by lean body mass does not bring further information into this investigation.

2- Specific Comments:

A- Reviewer’s comment: “Title: I think that the title of the article can be revised for more clarity. For example, I think that the ‘Psychophysiological responses’ can be changed to ‘Metabolic cost and perceptual responses’.”

A- Our Response: we thank the reviewer for this suggestion. However, to ensure terminological precision, we have opted for a slightly different revision. While "perceptual responses" typically refers to sensory experiences like perceived effort, our study also measured affective responses (pleasure and arousal) using the Feeling Scale and Felt Arousal Scale. Therefore, to accurately encompass all our measured psychological variables (both perceptual and affective), and to keep the terminological consistency for “energy cost”, we prefer keeping the title as it is.

B- Reviewer’s comment: “Abstract:

1) I think that the ‘psychophysiological responses’ can be changed (e.g., metabolic cost and perceptual responses).

2) I think that the ‘perceived effort (RPE)’ should be revised to ‘rating of perceived exertion (RPE)’.”

B- Our response:

1) Same response as (A) above

2) We thank the reviewer for this suggestion. However, to ensure terminological precision, we differentiate between “effort” and “exertion”. As mentioned in the Methodology section page 10, lines 193-196, based on (Abbiss et al., 2015; Venhorst et al., 2018a), “Exertion” is considered to refer to the peripheral fatigue (i.e., interoceptive sensory signals generated by the peripheral organs) while “Effort” is considered to refer to the mental fatigue (i.e., feedforward drive) to maintain lower limb ambulation (the sense of how hard one is trying). [Abbiss, C. R., Peiffer, J. J., Meeusen, R., & Skorski, S. (2015). Role of Ratings of Perceived Exertion during Self-Paced Exercise: What are We Actually Measuring? Sports Medicine (Auckland, N.Z.), 45(9), 1235–1243. https://doi.org/10.1007/S40279-015-0344-5 , Venhorst, A., Micklewright, D., & Noakes, T. D. (2018a). Towards a three-dimensional framework of centrally regulated and goal-directed exercise behaviour: a narrative review. British Journal of Sports Medicine, 52(15), 957–966. https://doi.org/10.1136/bjsports-2016-096907]

C- Reviewer’s comment: “Introduction:

1) I think that ‘psychological responses’ can be changed (e.g., perceptual responses).

2) I think that more detailed information regarding the ‘central pattern generators’ can be provided.”

C- Our Response:

1) Same response as (A) above

2) We added more details about central pattern generators on page 3-4, lines 54-57.

D- Reviewer’s comment: “Methodology:

1) What is the scientific rationale for the determination of the duration (i.e., 15 min)?

2) I think that the ‘Rating of Perceived Effort’ can be changed to ‘Rating of Perceived Exertion’.”

D- Our Response:

1) The 15-minute duration was chosen to allow participants to reach a physiological steady state and to provide sufficient time to observe the evolution of psychological responses similar to the following article: Ekkekakis, P., Hall, E. E., & Petruzzello, S. J. (2008). The relationship between exercise intensity and affective responses demystified: to crack the 40-year-old nut, replace the 40-year-old nutcracker! Annals of Behavioral Medicine : A Publication of the Society of Behavioral Medicine, 35(2), 136–149. https://doi.org/10.1007/S12160-008-9025-Z

2) Same response as (B) above.

E- Reviewer’s comment: “Results:

1) Please double-check the accuracy of the statistical methods (e.g., The dependent samples t-test (Page 13, Lines 260~265)).

2) It is not clear why PSF3 (not PSF2) condition was used to identify the influence of stride frequency on the measured variables.

E- Our Response:

1) We have double-checked these analyses. The dependent samples t-tests were used specifically to compare the stride frequencies between conditions (e.g., PSF3 vs. -10%PSF2) to confirm our experimental manipulation was successful. This is a manipulation check, separate from the main ANOVA analyses for the dependent variables. To clarify, we have revised the sentence to be more precise on page 12, lines 246-247.

2) Same response as in our response to general comments above.

F- Reviewer’s comment: “Discussion:

1) I think that most of the Discussion section is based upon speculations. I think that the article would be stronger if the authors could focus more on their research outcomes (e.g., relatively small difference in rating of perceived exertion). Furthermore, I think that the following article may possibly be useful for the interpretation/discussion regarding oxygen uptake and perceptual responses:

・Rose and Parfitt. Medicine and Science in Sports and Exercise, 40(10), 1852–1860, 2008.

2) I think that it may be interesting to present each participant’s felt arousal scale data.

3) I think that the information regarding the central pattern generators (Page 18, Lines 379~386) can be moved to the Introduction section.

4) I think that the ‘small sample size’ may not be related to the ‘generalizabil

Attachment Submitted filename: Response to Reviewers.docx

# Peer review: Decision Letter 1

**DOI:** 10.1371/journal.pone.0355226.r003

**Authors:** Luca Ardigò

14 May 2026

PONE-D-25-68663R1

Psychophysiological responses to stride frequency manipulation in men and women

PLOS One

Dear Dr. Basset,

Thank you for submitting your manuscript to PLOS ONE. After careful consideration, we feel that it has merit but does not fully meet PLOS ONE’s publication criteria as it currently stands. Therefore, we invite you to submit a revised version of the manuscript that addresses the points raised during the review process.

Please submit your revised manuscript by Jun 28 2026 11:59PM. If you will need more time than this to complete your revisions, please reply to this message or contact the journal office at plosone@plos.org. When you're ready to submit your revision, log on to https://www.editorialmanager.com/pone/ and select the 'Submissions Needing Revision' folder to locate your manuscript file.

Please include the following items when submitting your revised manuscript:

- A letter that responds to each point raised by the academic editor and reviewer(s). You should upload this letter as a separate file labeled 'Response to Reviewers'.
- A marked-up copy of your manuscript that highlights changes made to the original version. You should upload this as a separate file labeled 'Revised Manuscript with Track Changes'.
- An unmarked version of your revised paper without tracked changes. You should upload this as a separate file labeled 'Manuscript'.

If you would like to make changes to your financial disclosure, please include your updated statement in your cover letter. Guidelines for resubmitting your figure files are available below the reviewer comments at the end of this letter.

If applicable, we recommend that you deposit your laboratory protocols in protocols.io to enhance the reproducibility of your results. Protocols.io assigns your protocol its own identifier (DOI) so that it can be cited independently in the future. For instructions see: https://journals.plos.org/plosone/s/submission-guidelines#loc-laboratory-protocols. Additionally, PLOS ONE offers an option for publishing peer-reviewed Lab Protocol articles, which describe protocols hosted on protocols.io. Read more information on sharing protocols at https://plos.org/protocols?utm_medium=editorial-email&utm_source=authorletters&utm_campaign=protocols.

As the corresponding author, your ORCID iD is verified in the submission system and will appear in the published article. PLOS supports the use of ORCID, and we encourage all coauthors to register for an ORCID iD and use it as well. Please encourage your coauthors to verify their ORCID iD within the submission system before final acceptance, as unverified ORCID iDs will not appear in the published article. *Only*  the individual author can complete the verification step; PLOS staff *cannot*  verify ORCID iDs on behalf of authors.

We look forward to receiving your revised manuscript.

Kind regards,

Luca Paolo Ardigò, Ph.D.

Academic Editor

PLOS One

Journal Requirements:

If the reviewer comments include a recommendation to cite specific previously published works, please review and evaluate these publications to determine whether they are relevant and should be cited. There is no requirement to cite these works unless the editor has indicated otherwise.

Additional Editor Comments:

Please, carefully address point-to-point all Reviewer 1 and 2's issues (particularly Reviewer 2's).

[Note: HTML markup is below. Please do not edit.]

Reviewers' comments:

Reviewer's Responses to Questions

**Comments to the Author**

1. If the authors have adequately addressed your comments raised in a previous round of review and you feel that this manuscript is now acceptable for publication, you may indicate that here to bypass the “Comments to the Author” section, enter your conflict of interest statement in the “Confidential to Editor” section, and submit your "Accept" recommendation.

Reviewer #1: (No Response)

Reviewer #2: All comments have been addressed

Reviewer #3: All comments have been addressed

**********

2. Is the manuscript technically sound, and do the data support the conclusions?

The manuscript must describe a technically sound piece of scientific research with data that supports the conclusions. Experiments must have been conducted rigorously, with appropriate controls, replication, and sample sizes. The conclusions must be drawn appropriately based on the data presented.

Reviewer #1: Yes

Reviewer #2: Partly

Reviewer #3: Yes

**********

3. Has the statistical analysis been performed appropriately and rigorously?

Reviewer #1: I Don't Know

Reviewer #2: No

Reviewer #3: Yes

**********

4. Have the authors made all data underlying the findings in their manuscript fully available?

The PLOS Data policy requires authors to make all data underlying the findings described in their manuscript fully available without restriction, with rare exception (please refer to the Data Availability Statement in the manuscript PDF file). The data should be provided as part of the manuscript or its supporting information, or deposited to a public repository. For example, in addition to summary statistics, the data points behind means, medians and variance measures should be available. If there are restrictions on publicly sharing data—e.g. participant privacy or use of data from a third party—those must be specified.

Reviewer #1: Yes

Reviewer #2: Yes

Reviewer #3: Yes

**********

5. Is the manuscript presented in an intelligible fashion and written in standard English?

PLOS ONE does not copyedit accepted manuscripts, so the language in submitted articles must be clear, correct, and unambiguous. Any typographical or grammatical errors should be corrected at revision, so please note any specific errors here.

Reviewer #1: Yes

Reviewer #2: Yes

Reviewer #3: Yes

**********

6. Review Comments to the Author

Please use the space provided to explain your answers to the questions above. You may also include additional comments for the author, including concerns about dual publication, research ethics, or publication ethics. (Please upload your review as an attachment if it exceeds 20,000 characters)

Reviewer #1: It seems that the present version of manuscript has been improved.

Nevertheless, I still think that the ‘perceived effort (RPE)’ should be revised to ‘rating of perceived exertion (RPE)’.

Reviewer #2: Underpowered design for key claim: Sample size (n=19; 11 vs 8) is severely underpowered for between-sex comparisons, yet sex differences remain a central framing element. Even after revision, the inferential structure still leans on absence of evidence.

Conceptual overreach in conclusions: Claim that PSF “minimizes psychological responses” is imprecise and partially contradicted by results (valence increases rather than “responses” being minimized).

Ambiguity in construct definitions: Inconsistent distinction between “effort” and “exertion”; operationalization of RPE deviates from standard Borg usage without sufficient validation.

Methodological confounding (PSF2 vs PSF3): The imposed vs self-selected condition conflates stride frequency effects with cognitive load, attentional demand, and metronome-induced affective changes. This is not cleanly isolated.

Statistical design concerns:

Use of multiple paired t-tests risks inflated Type I error.

Justification for planned contrasts is post hoc and not transparently pre-registered.

Lack of correction strategy for multiple comparisons.

Effect size interpretation inconsistencies.

Measurement validity issues:

Ordinal affective scales treated with parametric summaries without justification.

Averaging across time (3–15 min) obscures temporal dynamics.

No reliability reporting for dual-observer stride counts.

Instrumentation inconsistency: Reported camera sampling rate error (250 Hz vs 120 Hz) indicates poor methodological control.

Insufficient control of confounders:

Menstrual cycle phase not biologically verified.

Psychological state influenced by metronome monotony not controlled.

No control for learning or adaptation effects across conditions.

Mechanistic speculation unsupported by data:

Claims about central command, CPG disruption, and neuromotor processes lack direct measurement.

Neurophysiological explanations exceed evidential basis.

Biomechanical neglect (partially corrected but still weak):

Insufficient integration of established biomechanical determinants of stride efficiency.

External validity limitations:

Homogeneous sample (young, healthy, active) undermines generalizability.

Overextension to exercise adherence and broader behavioral claims.

Data availability non-compliance risk:

Restricted dataset access may conflict with journal policy despite mitigation.

Clarity and reporting issues:

Terminological inconsistency (PSF1, PSF2, PSF3 logic not intuitively clear).

Figures and labels (e.g., NSF) previously inconsistent.

Some grammatical and phrasing errors persist.

Theoretical contribution limited:

Largely confirmatory of known U-shaped energy cost relationships.

Incremental contribution framed as broader psychophysiological insight without sufficient novelty.

Reviewer #3: The authors have thoroughly and satisfactorily responded to all of my feedback. I have no further concerns.

**********

7. PLOS authors have the option to publish the peer review history of their article (what does this mean?). If published, this will include your full peer review and any attached files.

If you choose “no”, your identity will remain anonymous but your review may still be made public.

**Do you want your identity to be public for this peer review?**  For information about this choice, including consent withdrawal, please see our Privacy Policy.

Reviewer #1: No

Reviewer #2: **Yes:**  Åke Elden

Reviewer #3: No

**********

[NOTE: If reviewer comments were submitted as an attachment file, they will be attached to this email and accessible via the submission site. Please log into your account, locate the manuscript record, and check for the action link "View Attachments". If this link does not appear, there are no attachment files.]

To ensure your figures meet our technical requirements, please review our figure guidelines: https://journals.plos.org/plosone/s/figures

You may also use PLOS’s free figure tool, NAAS, to help you prepare publication quality figures: https://journals.plos.org/plosone/s/figures#loc-tools-for-figure-preparation.

NAAS will assess whether your figures meet our technical requirements by comparing each figure against our figure specifications.

# Author comment: Author response to Decision Letter 2

**DOI:** 10.1371/journal.pone.0355226.r004

17 Jun 2026

Second Response to Reviewers

Second Revision

“Psychophysiological responses to stride frequency manipulation in men and women”

[ALREADY ADDRESSED] = done in the first revision  [NEW] = added in this revision  [FRAMING / SCOPE] = respectfully clarified

Dear Dr. Ardigò,

We thank you and the reviewers for the continued, constructive engagement with our manuscript. We are encouraged that Reviewer #1 considers the manuscript improved with a single remaining request, and that Reviewer #3 now states that we responded “thoroughly and satisfactorily” to all prior feedback with no further concerns. As you requested, we have given particular attention to Reviewer #2.

In reading Reviewer #2’s list, we noted that a number of the points concern issues that were already addressed in our first revision, and we respectfully draw attention to the relevant text rather than describing those changes as new. Reviewer #2’s comments fall into three groups: (i) concerns already addressed in the previous revision; (ii) matters of theoretical framing, which we have simplified to remove ambiguity; and (iii) requests that fall outside the scope of a single revision or of PLOS ONE’s publication criteria. We address every point below, and we have made several further additions this round to strengthen the manuscript.

Reviewer #1

Comment. It seems that the present version of the manuscript has been improved. Nevertheless, I still think that the ‘perceived effort (RPE)’ should be revised to ‘rating of perceived exertion (RPE)’.

Response. [NEW] We thank the reviewer for prompting greater precision. We agree, following Abbiss et al. (2015), that effort and exertion are related but slightly different constructs that are often used interchangeably and are difficult to separate with a single scale and standard instructions. We have made our usage conservative and consistent throughout: the instrument is now referred to as Borg's Rating of Perceived Exertion (RPE) scale, values are reported as RPE scores, and RPE is described in its standard sense as the perceived intensity of exertion/effort during the task. We have removed any claim that the scale measures a distinct "effort" or central-drive construct, as we did not collect the concurrent perceptual or neurophysiological measures that prior work has required to demonstrate such a dissociation.

Regarding the standardized instructions noted in our Methodology: we have retained them, because instruction wording is known to influence RPE (Abbiss et al., 2015). We have, however, clarified their purpose. The leg-focused prompt ("how do you perceive the difficulty in keeping your legs active at this moment?") was used to keep the perceptual referent constant across conditions, not as a means of measuring effort separately from exertion. The interpretation that the elevated RPE under imposed pacing may reflect central/cognitive demands now appears only in the Discussion, framed as a possible mechanism consistent with the psychobiological literature and as a direction for future work.

We are grateful for this suggestion, which also resolves the related construct-definition concern raised by Reviewer #2 (point 3).

Reviewer #2

We thank Reviewer #2 for the demanding review and have treated every item below. We note that the Reviewer indicated “all comments have been addressed,” and that several listed points were in fact addressed in our first revision; for those, we point to the existing text. Where a concern matches one raised by Reviewer #3 in the first round, we note that Reviewer #3 now regards it as fully resolved.

1. Statistical power and the sex comparison

Comment. Sample size (n = 19; 11 vs 8) is severely underpowered for between-sex comparisons, yet sex differences remain a central framing element. Even after revision, the inferential structure still leans on absence of evidence.

Response. [ALREADY ADDRESSED] Our first revision already added a post-hoc power analysis to the Limitations -with 11 men and 8 women, the minimum detectable between-group effect at 80% power and α = 0.05 is approximately Cohen’s d = 1.38, and power to detect d = 0.5 is only ~17% - and already states that the absence of significant sex differences must be read as “no difference detected,” not as equivalence (Limitations, [p. 25, lines 530-534]; Abstract , “in our sample”). [NEW] To remove any sense that sex remains a central claim, we have now relabelled the between-sex comparison explicitly as a secondary, exploratory aim (Introduction, aims paragraph, [lines 89]). Our primary hypothesis is tested within subjects, where the repeated-measures design retains adequate sensitivity; no further change to the sex comparison is possible without new data.

2. Precision of the central conclusion

Comment. Claim that PSF ‘minimizes psychological responses’ is imprecise and partially contradicted by results (valence increases rather than ‘responses’ being minimized).

Response. [ALREADY ADDRESSED] This wording was corrected in our first revision. The Abstract and Conclusion already state that the preferred stride frequency minimizes energy cost and RPE and is associated with higher positive valence: we no longer claim that psychological responses are “minimized” (Abstract, [p. 2, line 31]; Conclusion, [lines 547-548]). This also reflects the identical observation made by Reviewer #3 in the first round, which Reviewer #3 now considers resolved.

3. Construct definitions: effort vs. exertion and Borg usage

Comment. Inconsistent distinction between ‘effort’ and ‘exertion’; operationalization of RPE deviates from standard Borg usage without sufficient validation.

Response. [NEW] We thank the reviewer for prompting greater precision. We agree, following Abbiss et al. (2015), that effort and exertion are related but slightly different constructs that are often used interchangeably and are difficult to separate with a single scale and standard instructions. We have made our usage conservative and consistent throughout: the instrument is now referred to as Borg's Rating of Perceived Exertion (RPE) scale, values are reported as RPE scores, and RPE is described in its standard sense as the perceived intensity of exertion/effort during the task. We have removed any claim that the scale isolates a distinct "effort" or central-drive construct, as we did not collect the concurrent perceptual or neurophysiological measures that prior work has required to demonstrate such a dissociation.

Regarding the standardized instructions noted in our Methodology: we have retained them, because instruction wording is known to influence RPE (Abbiss et al., 2015). We have, however, clarified their purpose. The leg-focused prompt ("how do you perceive the difficulty in keeping your legs active at this moment?") was used to keep the perceptual referent constant across conditions, not as a means of measuring effort separately from exertion. The interpretation that the elevated RPE under imposed pacing may reflect central/cognitive demands now appears only in the Discussion, framed as a possible mechanism consistent with the psychobiological literature and as a direction for future work.

4. PSF2 vs. PSF3 — cognitive load, attentional demand, metronome effects

Comment. The imposed vs. self-selected condition conflates stride frequency effects with cognitive load, attentional demand, and metronome-induced affective changes. This is not cleanly isolated.

Response. [ALREADY ADDRESSED] [FRAMING / SCOPE] We would respectfully clarify that the PSF2-vs-PSF3 contrast is not a confound to be removed but is the comparison of interest: PSF2 and PSF3 are matched on stride frequency and walking speed, so the contrast isolates the effect of imposing an otherwise-preferred frequency. Our first revision already discussed the synchronization and attentional demands of metronome pacing as the leading candidate mechanism for the PSF2–PSF3 difference and noted this in the Discussion (Jäncke et al., 2000; Repp & Su, 2013; Moumdjian et al., 2018).

5. Statistical design: t-tests, planned contrasts, multiple comparisons, effect sizes

Comment. Use of multiple paired t-tests risks inflated Type I error. Justification for planned contrasts is post hoc and not transparently pre-registered. Lack of correction strategy for multiple comparisons. Effect size interpretation inconsistencies.

Response. [ALREADY ADDRESSED] [FRAMING / SCOPE] This concern is substantively identical to the one raised by Reviewer #3 in the first round; in the present round Reviewer #3 states that the statistical analysis has been addressed “thoroughly and satisfactorily” with “no further concerns.” For clarity: the primary inferential model is a repeated-measures (mixed) ANOVA, not a series of paired t-tests; paired t-tests appear only as a manipulation check on stride frequency, as already stated in the Methodology. The planned contrasts test an a priori set of theory-driven comparisons (PSF3 vs +10%PSF2; PSF3 vs −10%PSF2; PSF3 vs PSF2), and the rationale for choosing focused contrasts over omnibus post-hoc tests is already given in the first revision. PLOS ONE does not require pre-registration. [NEW] To address the multiple-comparison point directly, we have added a sensitivity analysis applying a Benjamini-Hochberg false-discovery-rate (FDR) correction to the family of planned contrasts within each primary outcome (Supporting Information, Table S1). After correction, the condition effects on V̇O₂ and on RPE remained significant for all contrasts (adjusted p ≤ .04), and the Feeling Scale contrasts remained significant at the conventional threshold (adjusted p ≈ .05). We note in the Results and Discussion that the valence effect, while surviving correction, is of more modest robustness than the metabolic and perceptual effects, and we have calibrated the valence wording accordingly.

6. Measurement validity: ordinal scales, time-averaging, inter-rater reliability

Comment. Ordinal affective scales treated with parametric summaries without justification. Averaging across time (3–15 min) obscures temporal dynamics. No reliability reporting for dual-observer stride counts.

Response. [NEW] (i) We agree this warranted explicit justification. We have added a statement to the Statistical Analysis section noting that, although the Feeling Scale, Felt Arousal Scale, and RPE yield ordinal ratings, parametric analysis follows established practice for affective and psychophysical scales in exercise science (Hardy & Rejeski, 1989; Ekkekakis et al., 2011) and is supported by evidence that parametric tests are robust to multi-category ordinal data (Norman, 2010). We also acknowledge the ordinal nature of these measures as a consideration in the Limitations. [ALREADY ADDRESSED] (ii) Temporal dynamics: time was already modelled as a within-subject factor in the ANOVA; we report the significant main effect of time on RPE (F (1.524, 25.901) = 16.349, p < 0.001) and the per-time-point values, so the time course is not lost. [NEW] (iii) Inter-rater reliability: stride counts were performed by two researchers who counted independently and blind to one another, and the mean of the two counts was used for each window specifically to minimize single-observer error. We have made this procedure explicit in the Methodology, lines 215-217.

7. Instrumentation: camera sampling rate

Comment. Reported camera sampling rate error (250 Hz vs 120 Hz) indicates poor methodological control.

Response. [ALREADY ADDRESSED] This was corrected in our first revision. The Methods already state that the Sony ZV-1M2 was operated at a sampling rate of 120 Hz (p. 10, line 204). the earlier 250 Hz value has not appeared since the original submission. We confirm that all stride-counting windows and analyses used this verified 120 Hz rate, and that the configuration does not affect any reported result.

8. Control of confounders: menstrual phase, monotony, learning effects

Comment. Menstrual cycle phase not biologically verified. Psychological state influenced by metronome monotony not controlled. No control for learning or adaptation effects across conditions.

Response. [ALREADY ADDRESSED] [FRAMING / SCOPE] (i) Menstrual phase: our first revision already states that we scheduled female participants in the follicular/non-active phase using a Menstrual Cycle Survey and a validated backward-count method (Schmalenberger et al., 2020), and already acknowledges in the Limitations that hormonal assays were not used and that some phase misclassification is possible (lines 547-550). Hormonal verification was beyond the scope and budget of this study; because the sex comparison is secondary, residual phase variability does not affect our primary within-subject conclusions. (ii) Metronome monotony: addressed under point 4 as part of the mechanism under study. (iii) Learning/adaptation: each condition was preceded by familiarization, sessions were separated by a 48-hour washout, and the four conditions were counterbalanced, as stated in the Methodology

9. Mechanistic interpretation (central command, CPG, neuromotor)

Comment. Claims about central command, CPG disruption, and neuromotor processes lack direct measurement. Neurophysiological explanations exceed evidential basis.

Response. [ALREADY ADDRESSED] We agree we did not measure neural activity directly. Our first revision already softened these passages with the consistent use of “may”, “likely” and “perhaps”. We retain them only as theoretically grounded interpretation.

10. Biomechanical determinants

Comment. Insufficient integration of established biomechanical determinants of stride efficiency.

Response. [ALREADY ADDRESSED] Our first revision already added a biomechanical discussion, presented alongside the neural account as a parallel, non-exclusive explanation for the increased cost and effort at non-preferred frequencies, drawing on the literature recommended in the first round (Holt et al., 1995; Hatsopoulos & Warren, 1996; Kuo, 2001; Russell & Apatoczky, 2016). The Reviewer notes this is “partially corrected”.

11. External validity and behavioral extrapolation

Comment. Homogeneous sample (young, healthy, active) undermines generalizability. Overextension to exercise adherence and broader behavioral claims.

Response. [ALREADY ADDRESSED] Our first revision already states in the Limitations that the findings generalize only to young, healthy, physically active adults, and in the Conclusion, we have already rephrased the adherence and behavioral statements as clearly labelled implications and directions for future research rather than demonstrated outcomes.

12. Data availability

Comment. Restricted dataset access may conflict with journal policy despite mitigation.

Response. [ALREADY ADDRESSED] We note that in the present round all three reviewers, including Reviewer #2, answered “Yes” to whether the data underlying the findings have been made fully available. As stated in our Data Availability Statement, the underlying dataset has been deposited at Mendeley Data under restricted access to protect participant anonymity, as mandated by our ethics approval; qualifying researchers may apply for access through the Health Research Ethics Board (T: +1 709 864 8871) subject to a Data Use Agreement. This arrangement follows the provisions of the PLOS Data policy for data subject to ethical restrictions, and we believe the concern is resolved.

13. Clarity and reporting (condition labels, figures, language)

Comment. Terminological inconsistency (PSF1, PSF2, PSF3 logic not intuitively clear). Figures and labels (e.g., NSF) previously inconsistent. Some grammatical and phrasing errors persist.

Response. [ALREADY ADDRESSED] The erroneous “NSF” label issue in the figures was corrected following the first round.

14. Theoretical contribution and novelty

Comment. Largely confirmatory of known U-shaped energy cost relationships. Incremental contribution framed as broader psychophysiological insight without sufficient novelty.

Response. [FRAMING / SCOPE] We respectfully note that PLOS ONE’s stated criteria base editorial decisions on scientific and methodological soundness rather than on perceived novelty or impact (PLOS ONE, Criteria for Publication; Editorial and Peer Review Process). We have accordingly directed our revisions toward rigo

Attachment Submitted filename: Second Response to Reviewers.docx

# Peer review: Decision Letter 2

**DOI:** 10.1371/journal.pone.0355226.r005

**Authors:** Luca Ardigò

24 Jun 2026

<div>PONE-D-25-68663R2

Psychophysiological responses to stride frequency manipulation in men and women

PLOS One

Dear Dr. Basset,

Thank you for submitting your manuscript to PLOS ONE. After careful consideration, we feel that it has merit but does not fully meet PLOS ONE’s publication criteria as it currently stands. Therefore, we invite you to submit a revised version of the manuscript that addresses the points raised during the review process.

==============================

Please, one last (hope) effort to address Reviewer 2's final remarks.

==============================

Please submit your revised manuscript by Aug 08 2026 11:59PM. If you will need more time than this to complete your revisions, please reply to this message or contact the journal office at plosone@plos.org. When you're ready to submit your revision, log on to https://www.editorialmanager.com/pone/ and select the 'Submissions Needing Revision' folder to locate your manuscript file.

Please include the following items when submitting your revised manuscript:

- A letter that responds to each point raised by the academic editor and reviewer(s). You should upload this letter as a separate file labeled 'Response to Reviewers'.
- A marked-up copy of your manuscript that highlights changes made to the original version. You should upload this as a separate file labeled 'Revised Manuscript with Track Changes'.
- An unmarked version of your revised paper without tracked changes. You should upload this as a separate file labeled 'Manuscript'.

If you would like to make changes to your financial disclosure, please include your updated statement in your cover letter. Guidelines for resubmitting your figure files are available below the reviewer comments at the end of this letter.

If applicable, we recommend that you deposit your laboratory protocols in protocols.io to enhance the reproducibility of your results. Protocols.io assigns your protocol its own identifier (DOI) so that it can be cited independently in the future. For instructions see: https://journals.plos.org/plosone/s/submission-guidelines#loc-laboratory-protocols. Additionally, PLOS ONE offers an option for publishing peer-reviewed Lab Protocol articles, which describe protocols hosted on protocols.io. Read more information on sharing protocols at https://plos.org/protocols?utm_medium=editorial-email&utm_source=authorletters&utm_campaign=protocols.

As the corresponding author, your ORCID iD is verified in the submission system and will appear in the published article. PLOS supports the use of ORCID, and we encourage all coauthors to register for an ORCID iD and use it as well. Please encourage your coauthors to verify their ORCID iD within the submission system before final acceptance, as unverified ORCID iDs will not appear in the published article. *Only*  the individual author can complete the verification step; PLOS staff *cannot*  verify ORCID iDs on behalf of authors.

We look forward to receiving your revised manuscript.

Kind regards,

Luca Paolo Ardigò, Ph.D.

Academic Editor

PLOS One

Journal Requirements:

If the reviewer comments include a recommendation to cite specific previously published works, please review and evaluate these publications to determine whether they are relevant and should be cited. There is no requirement to cite these works unless the editor has indicated otherwise.

Please review your reference list to ensure that it is complete and correct. If you have cited papers that have been retracted, please include the rationale for doing so in the manuscript text, or remove these references and replace them with relevant current references. Any changes to the reference list should be mentioned in the rebuttal letter that accompanies your revised manuscript. If you need to cite a retracted article, indicate the article’s retracted status in the References list and also include a citation and full reference for the retraction notice.

Additional Editor Comments:

Please, one last (hope) effort to address Reviewer 2's final remarks.

[Note: HTML markup is below. Please do not edit.]

Reviewers' comments:

Reviewer's Responses to Questions

**Comments to the Author**

1. If the authors have adequately addressed your comments raised in a previous round of review and you feel that this manuscript is now acceptable for publication, you may indicate that here to bypass the “Comments to the Author” section, enter your conflict of interest statement in the “Confidential to Editor” section, and submit your "Accept" recommendation.

Reviewer #1: All comments have been addressed

Reviewer #2: All comments have been addressed

**********

2. Is the manuscript technically sound, and do the data support the conclusions?

The manuscript must describe a technically sound piece of scientific research with data that supports the conclusions. Experiments must have been conducted rigorously, with appropriate controls, replication, and sample sizes. The conclusions must be drawn appropriately based on the data presented.

Reviewer #1: Partly

Reviewer #2: Yes

**********

3. Has the statistical analysis been performed appropriately and rigorously?

Reviewer #1: I Don't Know

Reviewer #2: Yes

**********

4. Have the authors made all data underlying the findings in their manuscript fully available?

The PLOS Data policy requires authors to make all data underlying the findings described in their manuscript fully available without restriction, with rare exception (please refer to the Data Availability Statement in the manuscript PDF file). The data should be provided as part of the manuscript or its supporting information, or deposited to a public repository. For example, in addition to summary statistics, the data points behind means, medians and variance measures should be available. If there are restrictions on publicly sharing data—e.g. participant privacy or use of data from a third party—those must be specified.

Reviewer #1: No

Reviewer #2: No

**********

5. Is the manuscript presented in an intelligible fashion and written in standard English?

PLOS ONE does not copyedit accepted manuscripts, so the language in submitted articles must be clear, correct, and unambiguous. Any typographical or grammatical errors should be corrected at revision, so please note any specific errors here.

Reviewer #1: Yes

Reviewer #2: Yes

**********

6. Review Comments to the Author

Please use the space provided to explain your answers to the questions above. You may also include additional comments for the author, including concerns about dual publication, research ethics, or publication ethics. (Please upload your review as an attachment if it exceeds 20,000 characters)

Reviewer #1: Regarding the data availability, it seems that the authors stated 'No - some restrictions will apply'.

Reviewer #2: The manuscript is scientifically sound and addresses an interesting research question. However, substantial concerns remain regarding interpretive overreach, unresolved conceptual confounding in the imposed pacing condition, overemphasis on underpowered sex comparisons, and excessive mechanistic inference unsupported by direct measurement. I therefore recommend major revision before publication.

The manuscript title overemphasizes sex comparisons despite the study being clearly underpowered for any meaningful between-sex inference.

The sample size (n = 19) remains insufficient for robust subgroup analysis, and sex-based conclusions remain more visible than statistically justified.

The manuscript continues to imply absence of sex differences when the design only permits absence of detected differences.

The theoretical framing around sex-specific biological variation is disproportionately emphasized relative to the weak inferential capacity of the dataset.

The distinction between primary and secondary study aims appears introduced reactively during revision rather than reflecting the original study design.

The PSF2 versus PSF3 comparison conflates stride frequency manipulation with externally imposed pacing and additional attentional or synchronization demands.

The manuscript does not convincingly isolate whether observed effects arise from biomechanical inefficiency or cognitive burden induced by metronome pacing.

The interpretation of PSF2 versus PSF3 effects still assumes causal specificity that the experimental design does not fully support.

The repeated invocation of central pattern generators (CPGs) introduces mechanistic explanations that were not directly measured in the study.

The discussion of central command mechanisms extends substantially beyond the empirical evidence collected.

Neurophysiological explanations remain speculative and should be more clearly separated from observed physiological findings.

The manuscript repeatedly infers neural regulatory disruption without any direct neurophysiological measurement.

The claim that preferred stride frequency reflects optimization of psychological as well as physiological processes remains conceptually overstated.

The argument that central command overrides inefficient motor patterns is theoretically interesting but empirically unsupported in this design.

The manuscript moves too quickly from observed physiological outcomes to higher-order explanatory models of motor control.

The novelty claim remains modest since U-shaped energetic cost relationships around preferred stride frequency are already well established.

The contribution beyond previous stride frequency literature remains incremental rather than conceptually transformative.

The study does not sufficiently distinguish replication of known physiological effects from genuinely novel psychophysiological insight.

The use of parametric statistical analysis on ordinal measures (Feeling Scale, Felt Arousal Scale, Borg RPE) remains methodologically debatable despite the provided justification.

The manuscript relies heavily on precedent literature to justify parametric treatment rather than demonstrating robustness within the present dataset.

Averaging repeated measurements across exercise periods may obscure important temporal fluctuations in affective responses.

The manuscript does not fully explore whether psychophysiological responses changed dynamically over time within conditions.

The metronome condition may independently influence affective valence through monotony or irritation, introducing uncontrolled psychological confounding.

The manuscript attributes increased RPE under imposed pacing primarily to exertional changes while alternative attentional explanations remain equally plausible.

The discussion occasionally blurs the distinction between perception of exertion and broader cognitive effort constructs.

The interpretation of elevated RPE as reflecting increased cognitive burden remains speculative and insufficiently validated.

The manuscript occasionally overinterprets associative findings as reflecting underlying causal mechanisms.

The discussion section remains more theoretically ambitious than warranted by a relatively simple experimental design.

Some causal language remains stronger than justified given the observational nature of several inferred mechanisms.

The link drawn between stride frequency manipulation and future exercise adherence remains weakly supported and conceptually premature.

Behavioral extrapolations regarding motivation and exercise adherence extend beyond what the experimental design directly tests.

The manuscript attempts to connect acute treadmill responses to broader behavioral decision-making without empirical evidence for such transfer.

The Data Availability Statement appears inconsistent, with contradictory references to restricted-access and fully public repositories.

The current data availability documentation does not clearly establish compliance with PLOS ONE’s strict open data requirements.

The manuscript still contains traces of revision-driven defensive framing, suggesting several methodological clarifications were added post hoc rather than being integral to the original study design.

Parts of the manuscript may still require harmonization.

The overall discussion remains overly mechanistic relative to the descriptive nature of the primary experimental findings.

The manuscript would benefit from a more conservative separation between empirical findings, plausible interpretations, and theoretical speculation.

The conceptual architecture of the paper exceeds the explanatory power of the experimental dataset.

The paper is scientifically sound overall, but interpretive ambition remains noticeably greater than evidential support.

**********

7. PLOS authors have the option to publish the peer review history of their article (what does this mean?). If published, this will include your full peer review and any attached files.

If you choose “no”, your identity will remain anonymous but your review may still be made public.

**Do you want your identity to be public for this peer review?**  For information about this choice, including consent withdrawal, please see our Privacy Policy.

Reviewer #1: No

Reviewer #2: **Yes:**  Åke Elden

**********

[NOTE: If reviewer comments were submitted as an attachment file, they will be attached to this email and accessible via the submission site. Please log into your account, locate the manuscript record, and check for the action link "View Attachments". If this link does not appear, there are no attachment files.]

To ensure your figures meet our technical requirements, please review our figure guidelines: https://journals.plos.org/plosone/s/figures

You may also use PLOS’s free figure tool, NAAS, to help you prepare publication quality figures: https://journals.plos.org/plosone/s/figures#loc-tools-for-figure-preparation.

NAAS will assess whether your figures meet our technical requirements by comparing each figure against our figure specifications.

# Author comment: Author response to Decision Letter 3

**DOI:** 10.1371/journal.pone.0355226.r006

14 Jul 2026

Luca Paolo Ardigò, Ph.D.

Academic Editor, PLOS ONE

Re: PONE-D-25-68663R2

“Psychophysiological responses to stride frequency manipulation in men and women”

Dear Dr. Ardigò,

Thank you for your continued and thoughtful handling of our manuscript, and for inviting a further revision. After carefully considering Reviewer #2’s most recent comments, we have concluded that the substantive issues raised are already addressed in the current version of the manuscript and that the remaining concerns reflect differences in interpretation rather than deficiencies that could be resolved through further revision. Accordingly, we respectfully resubmit the manuscript without additional substantive modifications and seek your guidance and editorial judgment on the most appropriate path forward.

To ensure that our position is fully transparent, we outline our reasoning below with specific reference to the relevant sections of the current manuscript.

We make this request with genuine respect for the peer-review process and for Reviewer #2, whose careful and rigorous evaluation has unquestionably strengthened the manuscript through successive rounds of review. Our concern is not with the review itself, but rather with the apparent limits of what further revision can realistically achieve. Given the current state of the manuscript and reviews, we believe it is appropriate to bring this matter to your attention and seek your guidance.

1. The reviewer has affirmed the manuscript’s soundness while maintaining a recommendation against publication.

In the current review round, Reviewer #2 explicitly confirmed that the manuscript is technically sound (Question 2: “Yes”) and that the statistical analyses are appropriate and rigorous (Question 3: “Yes”), while also indicating that all previous comments have been addressed. These are the core criteria on which PLOS ONE evaluates submissions. We therefore find ourselves in the unusual position of having a reviewer affirm the manuscript’s scientific and methodological soundness while continuing to recommend against publication.

We further note that Reviewer #2’s initial assessment recommended rejection on the basis of what were described as “fundamental methodological and interpretive flaws.” Although the reviewer's evaluation has evolved substantially over successive rounds—culminating in the present acknowledgment that the manuscript is technically sound, statistically rigorous, and responsive to prior comments—the recommendation against publication has remained unchanged. We respectfully submit that this apparent disconnect may now reflect a difference in interpretation or emphasis rather than unresolved concerns regarding scientific validity.

2. A recurring inconsistency between the formal assessment and the accompanying comments.

In the second review round, both Reviewers #2 and #3 indicated that all previous comments had been addressed; however, Reviewer #2 simultaneously provided an extensive new set of critiques. A similar situation has arisen in the current round: both Reviewer #1 and Reviewer #2 selected “all comments have been addressed,” yet Reviewer #2 again raised a new series of concerns.

We respectfully suggest that this pattern may indicate that the remaining comments stem less from a finite set of identifiable deficiencies and more from an ongoing difference in perspective regarding the manuscript’s interpretation and scope. While we have endeavoured to address each concern raised throughout the review process, the emergence of new critiques after prior comments have been deemed resolved has limited the degree of convergence that would ordinarily be expected over successive rounds of revision. As a result, the review process now appears to be centred on differing views regarding interpretation and emphasis rather than on unresolved methodological, analytical, or reporting issues.

3. The substantive points raised are already addressed in the current manuscript.

We wish to be specific in this regard, as we recognize that a decision not to make further revisions should be supported by a clear demonstration that the issues raised have already been addressed in the manuscript.

• Separation of interpretation from observation. The Discussion already presents the proposed neural and central-command mechanisms as hypotheses rather than established conclusions. The relevant passages explicitly acknowledge that these mechanisms are “not mutually exclusive” with biomechanical explanations, employ cautious language such as “we postulate,” and state—regarding the proposed central origin of perceived exertion—that this interpretation is offered “although we did not measure this directly.” In our view, the interpretive restraint requested by the reviewer is already embedded in the current text.

• The PSF2 versus PSF3 comparison. This distinction, including the possibility that metronome-paced running introduces additional attentional or synchronization demands, has been explicitly addressed through prior revisions. The Discussion identifies the “cognitive and neuromuscular demands of auditory–motor synchronization” as a potential contributing factor and interprets the PSF2 effect in relation to perceived autonomy and cognitive appraisal. Importantly, this clarification was originally requested by the other reviewers and was subsequently considered satisfactorily addressed.

• The sex comparison. The manuscript already identifies the between-sex analysis as a secondary objective, explicitly acknowledges that it is underpowered, provides the corresponding power calculation (minimum detectable effect size d ≈ 1.38; approximately 17% power to detect a medium effect), and cautions that the absence of statistically significant differences “should be interpreted cautiously and not as evidence of equivalence.” The findings are therefore presented with appropriate qualification and consistent with the limitations of the available sample size.

• Parametric analysis of ordinal scales. The manuscript already justifies the use of parametric analyses with reference to established practice in the literature (Hardy & Rejeski, 1989; Ekkekakis et al., 2011) and evidence regarding the robustness of such methods when applied to ordinal data (Norman, 2010). In addition, a Benjamini–Hochberg sensitivity analysis is reported in Supplementary Table S1, and the ordinal nature of the measures is explicitly acknowledged as a limitation. Reviewer #2 has also affirmed in the current round that the statistical analyses are appropriate and rigorous.

In our assessment, the additional changes implied by Reviewer #2’s comments would not address unresolved gaps in the manuscript, as the underlying issues are already covered in the current version. Rather, such changes would require substantial re-analysis and re-framing of the study, effectively transforming it into a different manuscript and moving beyond the version that Reviewers #1 and #3 have reviewed and endorsed. We respectfully consider that these changes would unlikely improve the scientific quality or clarity of the paper.

4. Two reviewers reached positive assessments of the same manuscript.

Reviewer #1 considers the manuscript substantially improved and, in the current round, indicated that all comments have been addressed. Similarly, Reviewer #3, in the previous round, concluded that all prior concerns had been satisfactorily resolved. As no issues remained outstanding from Reviewer #3’s evaluation, further consultation in the present round would not have been expected to yield additional feedback.

Consequently, the manuscript that Reviewer #2 continues to view critically is the same manuscript that two independent reviewers have assessed positively, including with respect to several of the specific issues that remain contested, such as the statistical analyses, the interpretation of the findings, and the distinction between the PSF2 and PSF3 conditions.

We also note that, in both the current and previous review rounds, Reviewer #2 indicated that all comments had been addressed. This repeated acknowledgment suggests that the concerns now being raised are not a continuation of unresolved prior issues, but rather reflect ongoing reservations regarding the interpretation, framing, or emphasis of the work. While we fully respect the reviewer's perspective, we respectfully submit that these concerns differ in nature from identifiable methodological, analytical, or reporting deficiencies that can be addressed through further revision.

Our request.

In light of Reviewer #2’s affirmation of the manuscript’s scientific soundness and statistical rigor, the positive assessments provided by Reviewers #1 and #3, and our view that the substantive issues raised have already been incorporated into the current version, we respectfully request that you consider making an editorial decision based on the evaluations of the reviewer panel as a whole. We consider that further rounds of evaluation focused on a single reviewer may unlikely yield additional convergence, given that the recommendation against publication has remained unchanged since the initial review despite the reviewer’s subsequent acknowledgment that the manuscript is technically sound, statistically rigorous, and adjust to prior comments. The remaining concerns appear to relate principally to matters of interpretation and emphasis rather than scientific validity.

We fully recognize that the final decision is yours, and we are grateful for the thoughtful and careful attention you have devoted to our manuscript throughout the review process, as reflected in your invitation to make a further effort at revision. We remain confident in the scientific soundness of the work, a view supported by both the formal review criteria and the assessments of two independent reviewers, and we would greatly appreciate your inputs regarding the most appropriate way forward.

We confirm that the manuscript is original, is not under consideration elsewhere, and that all authors have read and approved this submission. We declare no competing interests. Thank you for your time and consideration.

Sincerely,

The Authors

Attachment Submitted filename: Response_to_Reviewers_3rd re-submission.docx

# Editor report: Decision Letter 3

**DOI:** 10.1371/journal.pone.0355226.r007

**Authors:** Luca Ardigò

19 Jul 2026

Psychophysiological responses to stride frequency manipulation in men and women

PONE-D-25-68663R3

Dear Dr. Basset,

We’re pleased to inform you that your manuscript has been judged scientifically suitable for publication and will be formally accepted for publication once it meets all outstanding technical requirements.

Within one week, you’ll receive an e-mail detailing the required amendments. When these have been addressed, you’ll receive a formal acceptance letter and your manuscript will be scheduled for publication.

An invoice will be generated when your article is formally accepted. Please note, if your institution has a publishing partnership with PLOS and your article meets the relevant criteria, all or part of your publication costs will be covered. Please make sure your user information is up-to-date by logging into Editorial Manager at Editorial Manager® and clicking the ‘Update My Information' link at the top of the page. For questions related to billing, please contact billing support.

If your institution or institutions have a press office, please notify them about your upcoming paper to help maximize its impact. If they’ll be preparing press materials, please inform our press team as soon as possible -- no later than 48 hours after receiving the formal acceptance. Your manuscript will remain under strict press embargo until 2 pm Eastern Time on the date of publication. For more information, please contact onepress@plos.org.

Kind regards,

Luca Paolo Ardigò, Ph.D.

Academic Editor

PLOS One

Additional Editor Comments (optional):

Congratulations for the interesting work.

Reviewers' comments:

# Editor report: Acceptance letter

**DOI:** 10.1371/journal.pone.0355226.r008

**Authors:** Luca Ardigò

PONE-D-25-68663R3

PLOS One

Dear Dr. Basset,

I'm pleased to inform you that your manuscript has been deemed suitable for publication in PLOS One. Congratulations! Your manuscript is now being handed over to our production team.

At this stage, our production department will prepare your paper for publication. This includes ensuring the following:

* All references, tables, and figures are properly cited

* All relevant supporting information is included in the manuscript submission,

* There are no issues that prevent the paper from being properly typeset

You will receive further instructions from the production team, including instructions on how to review your proof when it is ready. Please keep in mind that we are working through a large volume of accepted articles, so please give us a few days to review your paper and let you know the next and final steps.

Lastly, if your institution or institutions have a press office, please let them know about your upcoming paper now to help maximize its impact. If they'll be preparing press materials, please inform our press team within the next 48 hours. Your manuscript will remain under strict press embargo until 2 pm Eastern Time on the date of publication. For more information, please contact onepress@plos.org.

You will receive an invoice from PLOS for your publication fee after your manuscript has reached the completed accept phase. If you receive an email requesting payment before acceptance or for any other service, this may be a phishing scheme. Learn how to identify phishing emails and protect your accounts at https://explore.plos.org/phishing.

If we can help with anything else, please email us at customercare@plos.org.

Thank you for submitting your work to PLOS ONE and supporting open access.

Kind regards,

PLOS ONE Editorial Office Staff

on behalf of

Prof. Luca Paolo Ardigò

Academic Editor

PLOS One
